# Many but not all pathogen-associated molecular patterns aggravate neurogenic heterotopic ossification after spinal cord injury

**DOI:** 10.1186/s12929-026-01237-y

**Published:** 2026-04-10

**Authors:** Selwin G. Samuel, Hsu-Wen Tseng, Bastien Rival, Valérie Barbier, Kavita Bisht, Marjorie Salga, Shrutika M Mate, Whitney Fleming, François Genêt, Sébastien Banzet, Jean-Pierre Lévesque, Dorothée Girard, Kylie A. Alexander

**Affiliations:** 1 Mater Research Institute-The University of Queensland, Translational Research Institute, 37 Kent Street, Woolloongabba, QLD 4102 Australia; 2 Department of Oral Pathology and Microbiology, Saveetha Dental College and Hospitals, Chennai, Tamil Nadu India; 3 Institut de Recherche Biomédicale des Armées (IRBA), INSERM UMR-MD 1197, 1, Rue du Lieutenant Raoul Batany, 92140 Clamart, France; 4 UPOH (Unité Péri Opératoire du Handicap), Physical and Rehabilitation Medicine Department, Raymond-Poincaré Hospital, Assistance Publique-Hôpitaux de Paris (AP-HP), Garches, France; 5 UR 20262 Handistart, UFR Simone Veil Santé, Versailles Saint-Quentin-en-Yvelines University (UVSQ), Paris Saclay University, Montigny le Bretonneux, France

**Keywords:** Neurogenic heterotopic ossification, Spinal cord injury, Infection, Pathogen-associated molecular patterns, Pattern recognition receptors, Inflammation, Interleukin-1, Oncostatin M, Bone formation

## Abstract

**Background:**

Neurogenic heterotopic ossifications (NHOs) are heterotopic bones that develop in periarticular muscles after traumatic brain (TBI) and spinal cord injuries (SCI). The mechanisms leading to NHO are incompletely understood and the only effective treatment to-date remains surgical resection. We previously established that several inflammatory pathways drive NHO pathogenesis in injured muscles in a mouse model of NHO and in humans. We also demonstrated a functional association between gram-negative bacterial infections and NHO development via lipopolysaccharide (LPS), a pathogen associated molecular pattern (PAMP), which exacerbated NHO in a Toll-like receptor-4 (TLR4)-dependent manner in mice.

**Methods:**

Using our mouse model of NHO induced by SCI and muscle injury in mice, we tested the effect of a large array of purified PAMPs post-surgery to mimic fungal, viral and bacterial infections and measured NHO bone volumes by micro-computerized tomography (microCT). The effect of PAMPs was also tested in vitro on human muscle progenitors and monocyte/macrophage populations.

**Results:**

Muscle progenitors and monocyte/macrophage populations from humans and mice express numerous pattern recognition receptors. In mice, numerous PAMPs produced by bacteria, viruses and fungi exacerbated NHO formation, and the majority of these PAMPs indirectly stimulated fibro-adipogenic progenitor (FAP) calcium mineralization in vitro via macrophages. Likewise, in humans, some PAMPs, particularly those binding to TLR2, directly and indirectly increased the calcium mineralization and osteogenic differentiation of human FAPs isolated from muscles surrounding human NHO. Finally, we established that the indirect stimulation of human FAP mineralization was mediated by inflammatory cytokines IL-1 and oncostatin M secreted by monocytes in response to PAMPs. Overall, our findings suggest that numerous types of infection have the potential to exacerbate NHO development and further highlight the role of oncostatin M and IL-1 signaling pathways in NHO pathophysiology.

**Supplementary Information:**

The online version contains supplementary material available at 10.1186/s12929-026-01237-y.

## Background

Neurogenic heterotopic ossifications (NHO) are pathological extra-skeletal bones that develop in periarticular muscles after severe injuries of the central nervous system (CNS) such as spinal cord injury (SCI), traumatic brain injury (TBI), cerebral stroke or anoxia [1–3]. The pathobiology of NHO is still incompletely understood [4] and the only curative treatment is surgical resection when pathological NHO are diagnosed [2, 5–7]. However, these surgeries can be very challenging as NHO often encase joints, large blood vessels and nerves [8, 9] and NHO can recur after surgery in 6% of cases [10]. The only preventive treatment that has shown some efficacy in retrospective studies is administration of cyclooxygenase inhibitors, suggesting that inflammation is an important component of NHO pathobiology [11].

In a mouse model of SCI-induced NHO where the spinal cord is transected between thoracic vertebrae T_11_ to T_13_ followed by a hindlimb muscle injury, we have demonstrated key roles of excessive inflammatory responses in injured muscles in response to SCI. Specifically, SCI triggers a spike of endogenous glucocorticoids produced by adrenal glands [12, 13] which causes abnormal overactivation of macrophages in injured muscles [14, 15], leading to excessive release of pro-inflammatory cytokines oncostatin M (OSM), interleukin-1 (IL-1)-α and IL-1β, which all directly contribute to the abnormal osteoblastic differentiation of mesenchymal fibro-adipogenic progenitors (FAPs) in injured muscles [15–17].

Several retrospective clinical studies have shown a significantly higher prevalence of NHO in SCI and TBI patients with inflammation (smokers, pressure ulcers, tracheostomy), polytrauma, or infections (pneumonia and urinary tract infections) [18–23]. Interestingly, heterotopic ossifications have also been shown to develop in patients with severe COVID-19, without any accompanying CNS injury [24–26].

Pattern recognition receptors (PRRs) are cell sensors that recognize conserved molecular structures produced by pathogens called pathogen-associated molecular patterns (PAMPs), as well as non-microbial signals (sterile inflammation) triggered by products released from cell or tissue damage, called damage-associated molecular patterns (DAMPs) [27, 28]. PRRs can be transmembrane proteins on plasma and endosomal membranes, or within the cytosol of immune and non-immune cells. PRRs recognize and bind their respective ligands to initiate downstream inflammatory and immune signaling pathways such as the release of inflammatory cytokines, and activation of the innate and acquired arms of the immune system through the NF-κB and interferon-signaling pathways [29–31]. However, the role of PRR-mediated signaling in NHO development had not been previously investigated until recently.

We previously identified a functional association between infections with gram-negative bacteria and NHO development [32]. Using our mouse model of SCI-NHO, we demonstrated that lipopolysaccharide (LPS), a PAMP purified from gram-negative *E. coli*, exacerbated NHO in a dose-dependent manner via the PRR toll-like receptor (TLR)-4 and the Toll-interleukin-1 receptor (TIR)-domain-containing adapter-inducing interferon-β (TRIF/TICAM1) adaptor [32]. In addition, a small case–control retrospective study in patients with TBI showed that infections with the gram-negative *Pseudomonas* species were significantly associated with NHO development [32]. Together, our data suggests that infection with gram-negative bacteria is an important factor contributing to NHO development. However, the potential role of other viral, fungal and other bacterial pathogens or the PAMPs they produce has not been investigated.

In this study, we tested a broad panel of bacterial, viral or fungal PAMPs for their potential to alter NHO development in vivo using our mouse model of SCI-induced NHO and determined whether these PAMPs altered the osteogenic potential of murine and human muscle progenitor cells in vitro.

## Methods

### Animals

All mouse experiments were approved by the University of Queensland Animal Ethics Committee (approval 2021AE000155) and by the US Department of Defense Animal Care and Use Review Office (approval SC200080.e001). In vitro experiments were performed with muscle progenitor cells sorted from 7 to 8-week-old C57BL/6 mice purchased from the Animal Resource Centre (Perth, Australia) or from an in-house C57BL/6 colony at the Translational Research Institute. All mice were housed at the Translational Research Institute, Biological Research Facility (Queensland, Australia) under specific pathogen-free conditions with ad libitum access to water and a standard diet chow (Specialty Feeds, Western Australia, Australia) and simulated 12-h light and dark cycles.

### SCI-NHO model

C57BL/6 female mice at 6–8 weeks old were anesthetized by intraperitoneal injection of 100 mg/kg ketamine and 10 mg/kg xylazine, and 1% isoflurane. A laminectomy was performed on the dorsal spine, and the spinal cord was trans-sectioned with a scalpel blade between vertebrae T_11_ and T_13_. Muscle and skin surrounding the transection were sutured with synthetically manufactured absorbable polydioxanone (Assut sutures; Cat# AS-MS4522F) and polyglycolic acid (SilverGlide; Cat# PG60417N) suture materials, respectively.

After surgery, while mice were still under the effect of anesthesia, the right hamstring muscle was injured via an intramuscular injection of cardiotoxin (CDTX) purified from *Naja pallida* venom (Latoxan: Cat# L8102) at 0.3125 mg/kg in a maximum volume of 50 μL for a 20 g mouse. After surgery, mice received a subcutaneous injection of 10 mg/kg ciprofloxacin (antibiotic) (2 mg/mL, Aspen) and 0.05 mg/kg buprenorphine for 3 days post-surgery for analgesia (0.3 mg/mL, Reckitt). Mice were also given Bactrim (800 mg/L, Roche) in drinking water as prophylaxis for bladder infections. As SCI causes paraplegia, bladders were expressed manually by gentle massage of the bladder, twice daily, throughout the experiments. The TLR2 inhibitor C29 (MedChem Express, HY-100461) was administered at 30 mg/kg in vehicle (saline, 20 mM sodium bicarbonate) via intraperitoneal (ip) injection immediately after surgery and then daily until day 3 post-surgery. While NHO development is similar in both males and females [12, 14], only females were used in this study to adhere to best ethical practice as bladder expression is easier in females, and the risk of bladder infections and rupture caused by prolonged bladder expression are therefore reduced compared to males [33].

### PAMPs

All purified PAMPs were purchased from Invivogen Inc (except for CpG phosphorothioate oligodeoxynucleotide ODN 1668 and its control GC-1668 which were purchased from Integrated DNA Technologies) and administered by injecting intramuscularly (i.m.) or i.p.. Supplemental Table S1 details all PAMPs used in this study.

### Measurement of NHO via microcomputed tomography (µCT)

Due to instrument upgrades during the course of this study, NHO was quantified in vivo or ex vivo using either the Inveon positron emission tomography/computed tomography (PET-CT) multimodality system (Siemens, Germany) as previously described [34] or the Molecubes β-Cube and X-Cube µPET-CT system (Molecubes) as previously described [12]. To quantify NHO volumes, the region of interest (ROI) was drawn around the muscles containing NHO, and these were then carefully checked from three dimensions to ensure adjacent long bones were not included in the ROI. Calcified NHO regions were defined as above the threshold of 450 Hounsfield units (HU). To avoid analysis bias, the samples were blinded before quantifying NHO volumes.

### Tissue collection

At 7 or 21 days after surgery, mice were euthanized by CO_2_ asphyxiation, and the right hind limbs were fixed in freshly made 4% paraformaldehyde (Sigma-Aldrich) at 4 °C for 24 h. For histologic analysis, bones were decalcified for at least two weeks in 14% EDTA (Astral Scientific), pH 7.2.

### Sorting of mouse muscle progenitors and leukocytes

To isolate murine skeletal muscle cells, hamstring muscles were harvested from naïve 5–6-week-old C57BL/6 mice and digested using a skeletal muscle dissociation kit (Miltenyi Biotec; Cat# 130–098-462) as per manufacturer’s instructions. For muscle leukocyte sorting only, hamstring muscles were injected with cardiotoxin (Latoxan; Cat# L8102) at 0.3125 mg/kg in PBS four days before harvest.

Muscle leukocytes were stained and sorted, using a BD FACSAria™ Fusion flow cytometer, with following monoclonal antibodies (Biolegend): FITC anti-mouse TER-119/Erythroid Cells (clone TER-119), FITC anti-mouse/human CD45R/B220 (clone RA3-6B2), FITC anti-mouse CD3ε (clone 145-2C11), PECy7 anti-mouse Ly-6G (clone 1A8), APC anti-mouse F4/80 (clone BM8), PE anti-mouse/human CD11b (clone M1/70), and Brilliant Violet 785™ anti-mouse CD45 (clone 30-F11). Mouse monocytes/macrophages were sorted as CD45^+^ Ter-119^−^ B220^−^ CD3ε^−^ CD11b^+^ Ly-6G^−^ F4/80^+^ cells.

Muscle progenitors were stained and sorted with following monoclonal antibodies (Biolegend): e660-rat anti-mouse CD34 (clone RAM34) and Brilliant Violet 785™ rat anti-mouse CD45 (clone 30-F11), FITC rat anti-mouse TER-119/Erythroid Cells (clone TER-119), FITC rat anti-mouse/human CD45R/B220 (clone RA3-6B2), FITC rat anti-mouse CD3ε (clone 145-2C11), FITC anti-mouse/human CD11b (clone M 1/70), FITC anti-mouse Gr-1 (clone RB6-8C5), PECy7 anti-mouse Sca1 (clone D7), and Brilliant Violet 421™ anti-mouse CD31 (clone 390). FAPs were sorted as CD45^−^, lineage (CD3ε, CD45R/B220, CD11b, Gr1, Ter119)-negative (Lin^−^) CD31^−^ Sca1^+^ CD34^+^, satellite cells as CD45^−^ Lin^−^ CD31^−^ Sca1^−^ CD34^+^ and endothelial cells as CD45^−^ Lin^−^ CD31^+^ Sca1^+^ as previously described [17].

### Isolation and RNA extraction of central bone marrow and spleen

RNA isolation of central bone marrow from C57BL/6 mice (positive control for PRR mRNA expression) was performed using femurs flushed with 1 ml Trizol (Life Technologies), followed by chloroform separation and GeneJET RNA cleanup and concentration micro kit (Thermo Fisher Scientific). For spleen, tissues were homogenized in 1 ml Trizol using a Fast Prep 24 homogenizer and Lysing Matrix D (MP Biomedicals) as per manufacturer’s instructions, followed by chloroform separation and RNA isolation using the Isolate II RNA Mini Kit (Bioline) as per manufacturer’s instructions. Reverse transcription of 1 μg of RNA was performed using the SensiFAST cDNA Synthesis Kit (ThermoFisher) as per manufacturer’s instructions.

### RNA extraction and qRT-PCR of mouse muscle progenitor cells

Cell populations of interest were directly sorted in TRIzol (Invitrogen, Cat# 10296028). mRNA was extracted from the sorted cells with the GeneJET RNA Cleanup and Concentration Micro Kit (Thermo Scientific; Cat# K0841). cDNA synthesis from 1 μg of the extracted mRNA was performed with SensiFAST™ cDNA Synthesis Kit (Meridian bioscience; Cat# BIO-65054). mRNA expression was analyzed using a single-step reverse transcription quantitative real-time polymerase chain reaction (qRT-PCR) using a ViiA 7 Real-Time PCR System (Life Technologies) using Taqman fast PCR Master Mix and TaqMan primer and fluorescent probe sets (listed in Tables S2 and S3) for 20 s at 95 °C, then 40 cycles at 95 °C (1 s) and 60 °C (20 s). Ct values were normalized by the expression of the housekeeping gene *Hprt* and presented as ratio to house-keeping gene (delta CT method). Quantification was performed using the QuantStudio™ 7 Flex Real-Time PCR System (Thermofisher Scientific; Cat# 4485701).

### Isolation and culturing of mouse bone marrow-derived macrophages (BMDMs)

Femurs from naïve C57BL/6 mice were flushed with 1 mL PBS + 2% newborn calf serum (NCS) using a syringe with a 23G needle and resuspended in 2 mL Eppendorf tubes. Cells were combined and seeded into a T-75 flask (Corning®, Cat# 430641U) with RPMI medium (Gibco Invitrogen; Cat# 12633012) containing 10% (v/v) fetal calf serum (FCS), 10 U/mL penicillin, 10 μg/mL streptomycin, 2 mM l-glutamine and 50 ng/mL recombinant mouse colony stimulating factor (CSF)-1 (BioLegend).

After 7 days of culture, when BMDMs reached 80–90% confluence, the media were aspirated, flasks were rinsed with PBS and cells were detached enzymatically using TrypLE™ Select (GIBCO Invitrogen, Cat# 12563-029) for 5 min at 37ºC with 5% CO_2_. Cells were then washed and centrifuged (at 370 g for 5 min at 4 °C), supernatants aspirated and cells were resuspended in 10 mL of fresh culture medium. The cells were seeded into two 24-well plates at a density of ~ 500,000 cells/well. PAMPs were added to BMDM cultures with either 20 ng/mL dipalmitoyl-Cys-Ser-(Lys)_4_ (Pam2CSK4), 200 ng/mL tripalmitoyl-Cys-Ser-(Lys)_4_ (Pam3CSK4), 10 μg/mL Zymosan, 200 ng/mL peptidoglycan from *S. aureus* (PGN-SA), 200 ng/mL N-acetylmuramyl-l-Ala-γ-d-Glu-d-meso-diaminopimelic acid (m-TriDAP), 200 ng/mL Furfurman, 200 ng/mL Gardiquimod, 200 ng/mL 6-O-(2'(R,S)-tetradecyloctadecanoyl)-d-glucose (GlcC14C18), 200 ng/mL flagellin purified from *Pseudomonas aeruginosa*, 200 ng/mL polyinosinic:polycytidylic acid (poly(I:C)) or without any PAMP (control) and further cultured at 37 °C with 5% CO_2_ for 18 h. After stimulation, the conditioned supernatants from individual wells were collected without disturbing the adherent cell surface, centrifuged and stored at − 30 °C until further use.

### Mouse FAP culture, osteogenic differentiation, and quantification

FAPs were isolated from naïve hamstring muscles as described above (CD3ε, CD45R/B220, CD11b, Gr1, Ter119)-negative CD45^−^ CD31^−^ Sca1^+^ CD34^+^) and were sorted directly into αMEM with 20% FCS, and subsequently seeded into T25 flasks in DMEM with high glucose (GIBCO Invitrogen, Cat# 11960-044) containing 20% (v/v) FCS, 10% horse serum, 10 U penicillin/mL, 10ug/mL streptomycin and 2 mM l-glutamine. When cells reached 80–90% confluence, the media were aspirated, flasks were rinsed in 1X PBS and cells were detached enzymatically using TrypLE™ Select (GIBCO Invitrogen, Cat# 12563-029) for 5 min at 37ºC with 5% CO_2_. Cells were then washed and centrifuged (at 370 g for 5 min at 4 °C), supernatants aspirated and cells resuspended in 1 mL of fresh culture medium. Cell counts and viability were performed using a 1:1 dilution (v/v) with 0.4% (w/v) Trypan Blue stain (GIBCO Invitrogen, Cat# 15250-061). Cells were then seeded into a sterile 96-well plate at a density of 4000 cells/cm^2^ and further cultured in growth medium until confluent.

To induce osteogenic differentiation, growth medium was aspirated and replaced by osteogenic medium (α-MEM supplemented with 10% FCS, 10 U/mL penicillin, 10 μg/mL streptomycin, 10 mM β-glycerophosphate, 200 μM phosphoascorbic acid, 2 mM CaCl_2_, and 0.2 μM dexamethasone), in the absence or presence of increasing concentrations of PAMPs or 10% (v/v) BMDM-conditioned media following PAMP stimulation as described above. In control wells, the growth medium was replaced by control medium (α-MEM supplemented with 10% (v/v) FCS). After 12 days of incubation at 37 °C with 5% CO_2_, plates were fixed and quantification of calcium mineralization was measured via alizarin red staining [16] with data represented as normalized absorbance values at 562 nm relative to osteogenic medium alone wells.

### Isolation of human fibro-adipogenic progenitors (hFAPs) from NHO biopsies

After obtaining informed consent from patients, NHO biopsies were collected from surgical waste following their excision from patients with TBI or SCI at Raymond Poincaré Hospital (Garches, France). Muscle fragments were excised from around the NHO mass and minced using scalpel and small scissors and incubated in 1.5 mg/ml pronase (Sigma-Aldrich) in α-MEM for 45 min in a 37 °C water bath. After addition of α-MEM supplemenSted with 15% FCS, 10 U/mL penicillin, 10 μg/mL streptomycin, the cell suspension was filtered through a 100 µm cell strainer (BD Falcon) followed by a 40 µm cell strainer (BD Falcon). Isolated muscle progenitor cells (MPCs) were maintained for 10 days in α-MEM supplemented with 15% FCS, 1% P/S and 10 ng/ml basic fibroblast growth factor (FGF) (R&D Systems). Human MPCs were trypsinized and incubated for 30 min with biotinylated anti-human PDGFRα (Cat# BAF322, R&D Systems) goat polyclonal antibody and CD56-PE (clone B159, BD Pharmigen) monoclonal antibody in PBS 2% FCS, 2 mM EDTA or with control isotypes IgG1 PE (Cat# A07796, Beckman Coulter) and biotinylated goat IgG (Cat# BAF108, R&D Systems). Cells were washed and incubated for 30 min with Streptavidin-APC/Cy7 (Sony) and 7-AAD viability dye (Sony). Cells were washed and filtered through a 30 µm cell strainer (Sysmex) and sorted using FACSAria III SORP sorter (BD Biosciences). Sorted PDGFRα^+^ CD56^−^ hFAPs were seeded at 3,000 cell /cm^2^ in α-MEM supplemented with 10% FCS and 1% P/S.

### Isolation of human CD14^+^ monocytes and preparation of conditioned medium (CM)

Human CD14^+^ blood monocytes were isolated from whole blood donation buffy coats from the Centre de Transfusion Sanguine des Armées (Clamart, France). All donors gave their consent for research use of residues not used in the preparation of blood products. PBMC were recovered using Ficoll gradient (Pan-Biotech). CD14^+^ monocytes were then isolated following magnetic separation using CD14 microbeads (Miltenyi Biotec). CD14^+^ cells were seeded at 1.25 × 10^6^ cells per cm^2^ and stimulated with either 200 ng/ml Pam2CSK4; 200 ng/ml Pam3CSK4; 100 ng/ml LPS (LPS 100); 200 ng/ml M-TriDAP; 200 ng/ml flagellin, 200 ng/ml ODN 2395 (CpG); 200 ng/ml Furfurman; 200 ng/ml Zymosan (Zymosan 200); 10 µg/ml Zymosan (Zymosan 10 000); 200 ng/ml Gardiquimod; 200 ng/ml Trehalose-6,6-dibehenate (TDB); 200 ng/ml LPS (LPS 200); 200 ng/ml Poly(I:C), lipofectamine or 200 ng/ml Poly(I:C) + lipofectamine, 100 ng/ml LPS (L6529, Sigma-Aldrich), 200 ng/mL Pam2CSK4, 200 ng/mL Pam3CSK4 or no stimulation. Conditioned media were collected, centrifuged for 5 min at 500 g and used at 10% (v/v) in cell culture medium during osteogenic differentiation assays.

### In vitro osteogenic differentiation assay and mineralization quantification of hFAPs

hFAPs were seeded at 3000 cells per cm^2^ in α-MEM with 10% FCS and 1% P/S. After 3 days, the culture medium was replaced by control medium (α-MEM with 10% FCS 1% P/S) or osteogenic medium (α-MEM 10% FCS 1% P/S, 0.052 mg/mL dexamethasone, 12.8 µg/mL ascorbic acid and 2.15 mg/mL β-glycerophosphate (Sigma-Aldrich) for 12–14 days. PAMPs were directly incubated with hFAPs at the indicated concentrations or CM at 10% (v/v). For OSM and IL-1 inhibition assays, hFAPs were incubated for 2 h with IL-1RA (200-01RA, Peprotech) at 500 ng/ml prior to CM addition. Neutralizing mouse anti-human OSM monoclonal antibody (MAB295, Clone 17,001, Bio-Techne) or mouse IgG2a isotype control (Cat# MAB003, Clone 20,102, Bio-Techne) were used at 10 µg/ml with a 2-h pre-incubation in CM prior to addition to hFAPs. At the end of the differentiation process, quantification of mineralization was performed using Alizarin Red S staining [32] with data represented as normalized absorbance values at 405 nm relative to osteogenic medium alone wells.

### Quantification of cytokines in human CD14^+^ blood monocyte supernatants

OSM level was evaluated using Human OSM DuoSet ELISA Kit (Cat# DY295, R&D Systems) according to manufacturer’s instructions. Human IL-1α and IL-1β quantification were performed using LEGENDplex™ Human Inflammation Panel 1 (13-plex) (BioLegend) according to manufacturer’s instructions.

### Human cells RNA extraction and RT-qPCR

Total RNA was extracted using QIAzol lysis reagent (Qiagen) and chloroform (Sigma-Aldrich) extraction. RNA precipitation was performed using isopropanol (Sigma-Aldrich) and GlycoBlue (Ambion) for 20 min at − 20 °C. Pellet were washed with 75% ethanol before RNAse free water resuspension. Total RNA concentrations were evaluated by NanoDrop (ThermoFisher), pre-diluted to 20 ng/µL for mRNA and stored at − 80 °C. Reverse transcriptions were performed with RT^2^ First Strand kit (Qiagen). Post-RT cDNA were diluted to 1:80 before RT-qPCR amplification using Quantitect SYBR Green and Quantitect primers (Qiagen) (Table S2). Three reference genes were selected (FAPs: *HPRT*, *RPLP0*, *PPIA* and CD14^+^: *ACTB*, *GAPDH*, *PPIA*) using Genorm (v3.4) and normalized by the geometric mean of 1.9^(ΔCt to reference gene).

### Western blots

hFAPs in osteogenic assays were washed in PBS before cell lysis incubation using PBS, Triton X-100 1%, NP40 1%, sodium dodecyl sulphate 0.1%, deoxycholic acid 0.5% and protease inhibitors cocktail (Cat#11697498001, Roche) buffer. Cells were scraped and incubated 20 min on ice. Supernatants were collected after centrifugation (at 13,000×g for 15 min at 4 °C). Protein concentrations were quantified using Pierce BCA protein assay kit (23,252, Thermo Fisher Scientific) following the manufacturer’s instructions. 20 µg protein were loaded for electrophoresis in 10% acrylamide SDS-PAGE gels (161–0182, BioRad) before transfer on PVDF membrane (Trans-Blot Turbo, BioRad). Membranes were saturated for 2 h with 5% non-fat dry milk and subsequently incubated with specific anti-RUNX2 antibody (1/1000, ab192256, Abcam) at 4 °C overnight. Membranes were probed with HRP secondary antibody (1/200,000, ab205718, Abcam) at room temperature for 2 h. Enhanced ChemiLuminescence incubation (ECL, 170–5061, Bio-Rad), imaging and densitometric analysis were performed on ChemiDoc XRS + (Bio-Rad) using ImageLab for whole membrane Stain-Free normalization.

### Statistical analysis

Data are presented as mean and standard deviation. Statistically significant differences of two groups were calculated by the non-parametric Mann–Whitney test or by 2-tailed Student’s t test. For more than two groups, statistical significances were determined using one-way or two-way analysis of variance (ANOVA) or non-parametric Kruskal–Wallis test depending on distribution normality. All tests were performed using PRISM 8 software (GraphPad, La Jolla, CA).

## Results

### PRR expression by mouse and human skeletal muscle cells

We have previously reported that LPS purified from gram-negative bacteria exacerbates NHO development in mice via its receptor TLR4 [32]. Interestingly, TLR4 was expressed by numerous cells within the muscle that contribute to muscle regeneration and/or NHO development [17] such as satellite cells (SCs), FAPs and monocytes/macrophages (MΦ). To investigate whether additional PRRs are expressed by these cells, PRR mRNA expression was quantified by qRT-PCR in sorted mouse and human muscle progenitor cells (including SCs, FAPs and endothelial cells) and monocyte/macrophage populations.

**Fig. 1:**
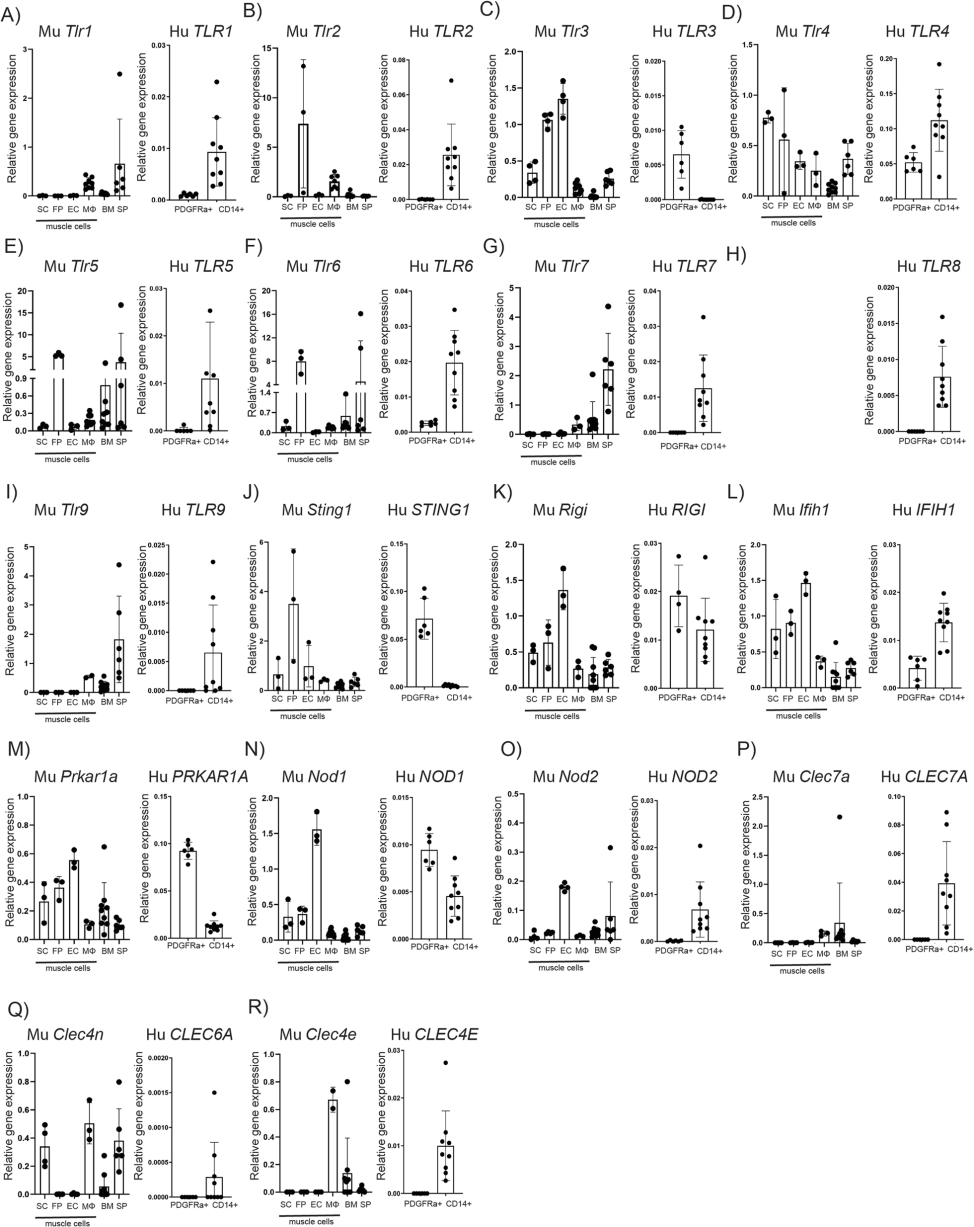
Expression of PRRs by mouse and human skeletal muscle cells. Expression of PRR mRNA transcripts in mouse satellite cells (SC), FAPs (FP), endothelial cells (EC) and monocyte/macrophages (MΦ) sorted from skeletal muscles. Whole mouse bone marrow (BM) cells and splenocytes (SP) were used as positive controls for these transcripts. Human PDGFRα^+^ FAPs were sorted from muscles surrounding NHO biopsies, and CD14^+^ monocytes were isolated from peripheral blood from healthy donors. **A** TLR1, **B** TLR2, **C** TLR3, **D** TLR4, **E** TLR5, **F** TLR6, **G** TLR7, **H** TLR8, **I** TLR9, **J** STING1, **K** RIGI, **L** MDA5 (IFIH1), **M** PRK, **N** NOD1, **O** NOD2, **P** Dectin-1 (CLEC7A), **Q** Dectin-2 (Clec4n/CLEC6A), **R** Mincle (CLEC4E). For each gene, left hand histogram represents expression of the mouse gene in mouse cells and the right-hand histogram represents expression of the human gene in human cells. For mouse cells, relative mRNA expression was quantified relative to house-keeping gene *Hprt*. For human cells, values were normalized using three references genes *HPRT*, *RPLP0* and *PPIA* for FAPs and *ACTB*, *GAPDH* and *PPIA* for CD14^+^ cells. Each dot represents a mouse or a human donor, bars represent mean ± SD

PRR expression in mouse muscle cells was heterogeneous among the cell types. All PRRs were expressed by MΦ isolated from skeletal muscles albeit at varying levels (Fig. 1) with mouse bone marrow cells and splenocytes used as positive controls for mouse PRR expression. Interestingly, some PRRs were detected in all cells isolated from mouse muscle such as *Tlr3*, *Rigi* (also called RIG-I), *Sting1*, *Ifih1* (MDA-5), *Prkar1a* (PKR), *Nod1* and *Nod2*. Finally, *Tlr1*, *Tlr7*, *Tlr9*, *Clec7a* (Dectin-1) and *Clec4e* (Mincle—macrophage inducible Ca2^+^-dependent lectin receptor) mRNA were largely undetectable in muscle progenitors.

*TLR4, TLR6, NOD1, PRKAR1A*, *IFIH1* (MDA-5) and *RIGI* were expressed in both human muscle hFAPs isolated from NHO biopsies and CD14^+^ cells isolated from human blood (Fig. 1). *TLR3* expression was restricted to hFAPs whereas *TLR7*, *TLR8*, *TLR9*, *NOD2*, *CLEC7A* (Dectin-1), *CLEC6A* (Dectin-2) and *CLEC4E* (Mincle) were restricted to CD14^+^ monocytes. Overall, these data show that PRRs are expressed in mouse and human immune and non-immune cells in muscles (Results from Fig. 1 are summarized in Table S3) and therefore have the potential to respond to PRR ligands and trigger downstream cellular responses.

### Most but not all PAMPs exacerbate SCI-NHO development in vivo in mice

As most PRRs investigated were expressed by one or several cell populations in mouse muscles, we then undertook a horizontal analysis to test a large array of PAMPs produced by, or mimicking PAMPs produced by either bacteria, fungi or viruses, which bind these PRRs. To this end, purified PAMP agonists for a wide variety of TLRs (TLR1/2 and TLR2/6 complexes, TLR3, TLR6, TLR7, TLR9), C-type lectin receptors Dectin-1, Dectin-2 and Mincle, and nucleotide-binding oligomerization domain (NOD)-like receptors NOD1 and NOD2 (Table S1) were injected into mice that underwent SCI and a CDTX-induced hamstring muscle injury to test their ability to alter NHO development.

**Fig. 2:**
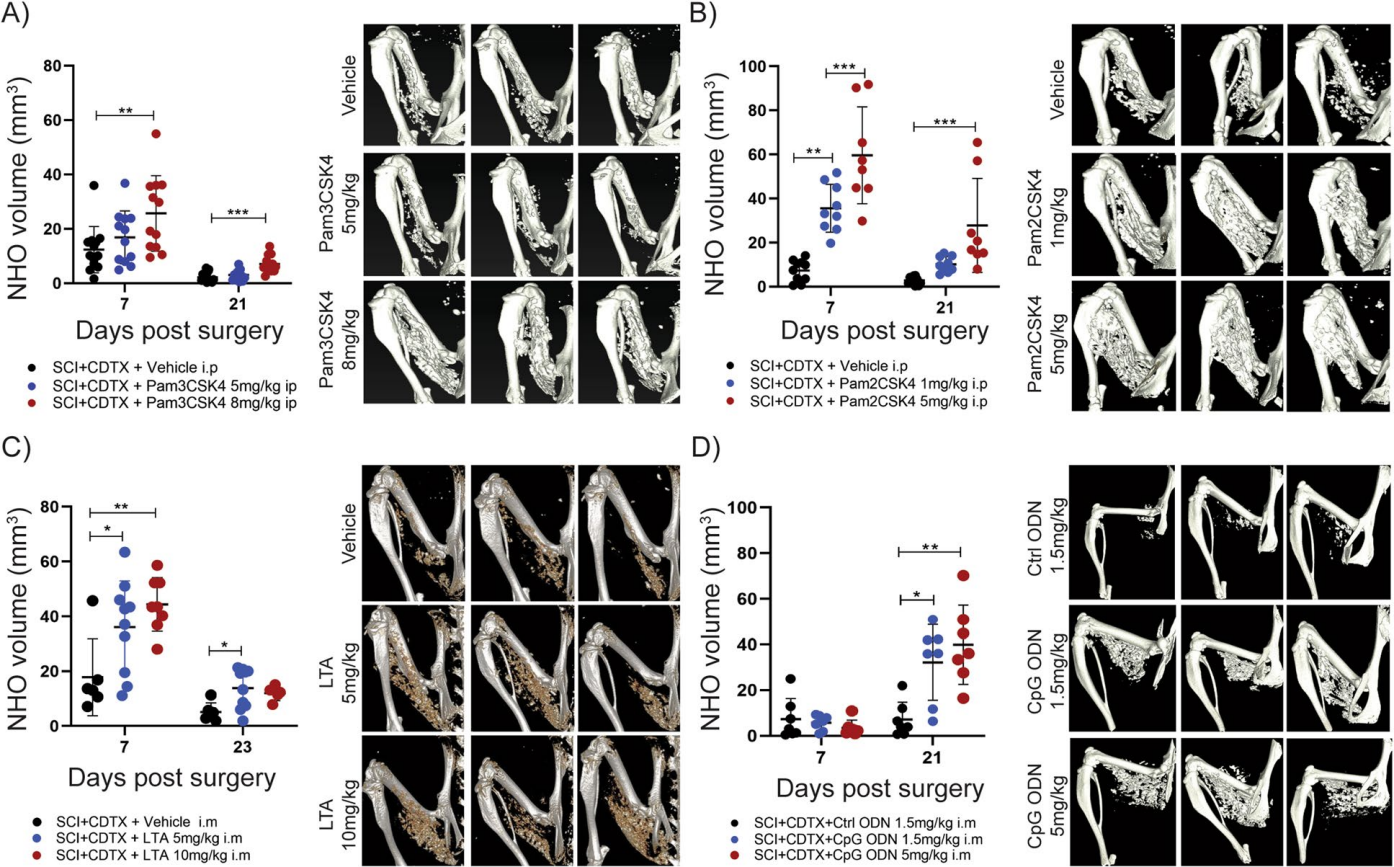
TLR agonists mimicking bacterial PAMPs exacerbate SCI-NHO in mice. C57BL/6 mice underwent SCI plus muscle injury via an intramuscular injection of CDTX. **A** NHO bone volumes at days 7 and 21 in mice treated post-surgery with vehicle or Pam3CSK4 (5 or 8 mg/kg i.p.), with representative μCT images at 7 days post-surgery. **B** NHO bone volumes at days 7 and 21 in mice treated post-surgery with vehicle or Pam2CSK4 (1 or 5 mg/kg i.p.), with representative images 7 days post-surgery. **C** NHO bone volumes at days 7 and 23 in mice treated post-surgery with vehicle or LTA (5 or 10 mg/kg i.m.) mixed with CDTX at the time of surgery with representative images 7 days post-surgery. **D** NHO bone volumes at days 7 and 21 in mice administered control ODN or CpG ODN1668 (1.5 or 5 mg/kg i.m.) mixed with CDTX at the time of surgery, with representative μCT images 7 days post-surgery. Each dot represents an individual mouse, bars represent mean ± SD, one-way ANOVA. *p < 0.05, **p < 0.01, ***p < 0.001

TLR agonists mimicking bacterial PAMPs, specifically the synthetic lipopeptides Pam3CSK4 (Fig. 2A) and Pam2CSK4 (Fig. 2B) which activate TLR1/TLR2 [35] and TLR2/TLR6 [36] heterodimers respectively, and lipoteichoic acid (LTA) (Fig. 2C) which activates both TLR2 heterodimers [37]. Finally, the single stranded CpG phosphorothioate oligodeoxynucleotide ODN1660 (Fig. 2D) which activates TLR9 in a sequence specific manner [38]. These PAMPs or appropriate controls, were administered to mice either locally (via an intramuscular injection) or systemically (via intraperitoneal injection) immediately after surgery, depending on how these PAMPs were best tolerated. Micro-computed tomography at 7- and 21-days post-surgery demonstrated that all TLR agonists exacerbated NHO in a dose-dependent manner. While exacerbation of NHO volumes was particularly evident 7 days post-surgery for Pam3CSK4, Pam2CSK4 and lipoteichoic acid and was similar to LPS [32], the effect of ODN1660 was delayed with increased NHO volumes by day 21 post-surgery. Of note, flagellin purified from *P. aeruginosa* flagella, a potent agonist of TLR5 at the surface of immune cells [39] and cytosolic NOD-like receptors NLRC4 and NAIP5 [40], were the exception with no effect on NHO bone volumes even at the highest dose tolerated by mice (Fig. S1).

**Fig. 3:**
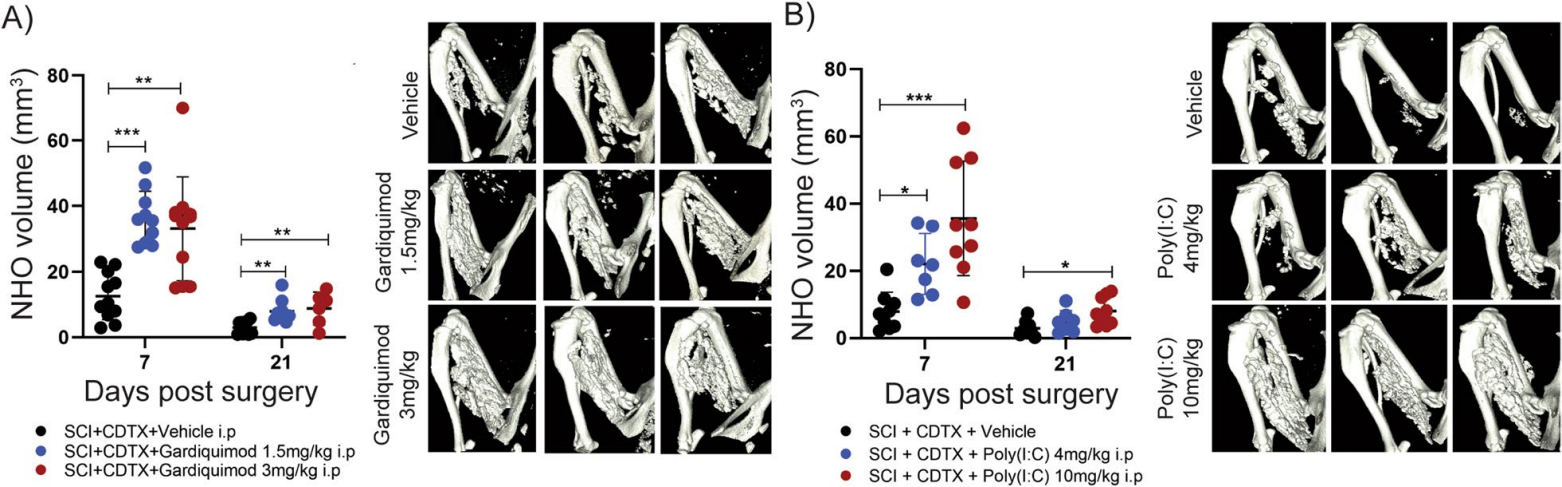
TLR agonists mimicking viral PAMPs exacerbate SCI-NHO in mice. C57BL/6 mice underwent SCI plus muscle injury via an intramuscular injection of CDTX (0.3125 mg/kg). **A** NHO bone volumes at days 7 and 21 in mice treated post-surgery with vehicle or gardiquimod (1.5 and 3 mg/kg i.p.), with representative μCT images at 7 days post-surgery. **B** NHO bone volumes at days 7 and 21 in mice treated post-surgery with vehicle or Poly(I:C) (4 and 10 mg/kg i.p.) at 6, 48 and 96 h after SCI, with representative μCT images at 7 days post-surgery. Each dot represents an individual mouse, bars represent mean ± SD, one-way ANOVA. *p < 0.05, **p < 0.01, ***p < 0.001

We next tested the effects of TLR agonists that mimic viral PAMPs such as the endosomal TLR7 agonist gardiquimod, a synthetic imidazoquinoline that mimics GU-rich motifs found in single stranded viral RNA [41], and the double stranded RNA mimic poly(I:C) which activates endosomal TLR3 [42] as well as the two cytosolic helicases RIG-I and MDA5/IFIH1 [43] and RNA-activated protein kinase PKR [44]. Gardiquimod was administered intraperitoneally immediately after surgery, whereas an adapted dosage regimen was required for poly(I:C) (6, 48 and 96 h post-surgery). This adapted regimen was needed as poly(I:C) receptors are all intracellular [43] and poly(I:C) is a polyanion than does not passively cross the negatively charged plasma membrane of mammalian cells. Both gardiquimod and poly(I:C) significantly increased NHO volumes in a dose-dependent manner (Fig. 3A, B).

**Fig. 4:**
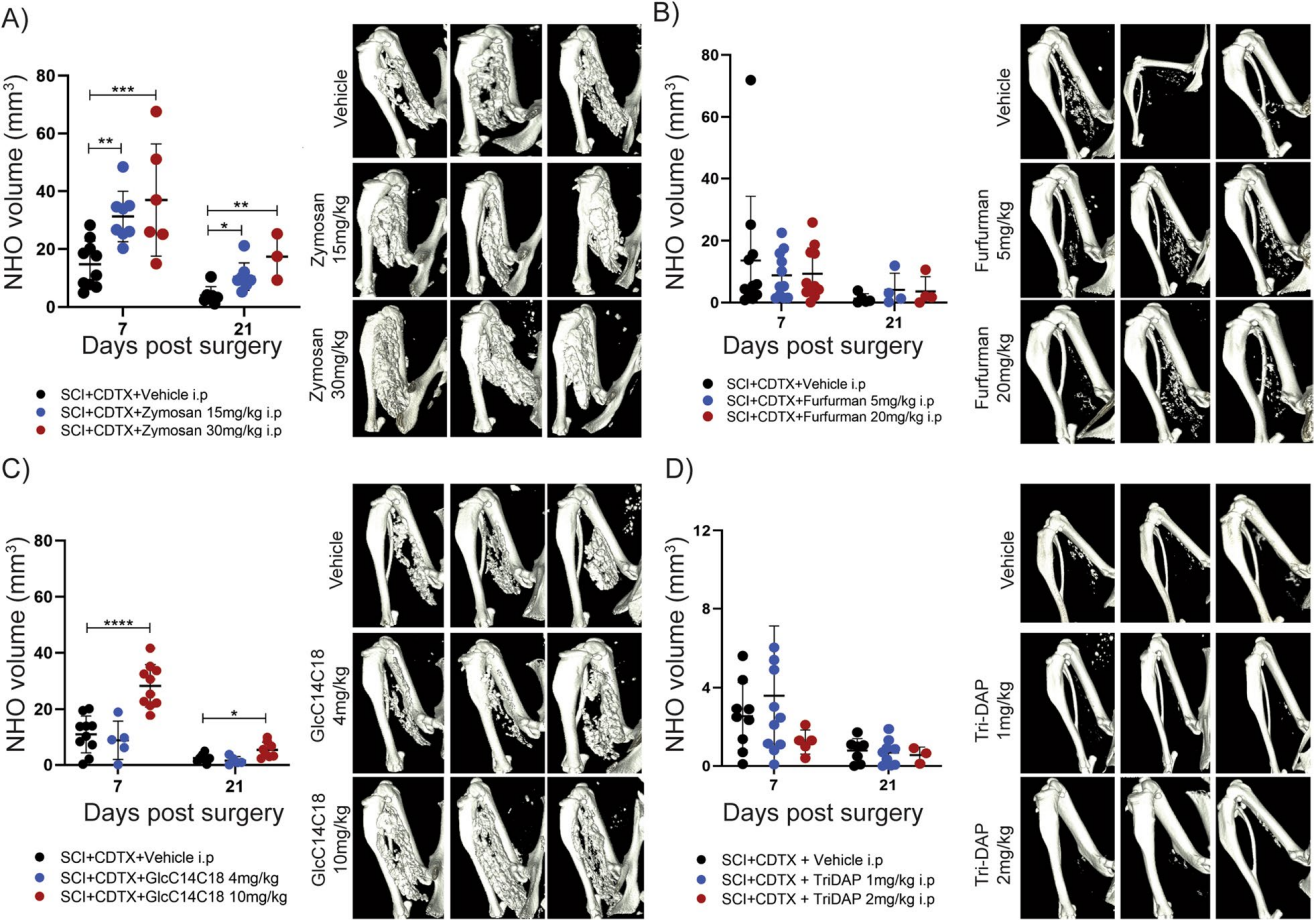
Effect of yeast and mycobacterial PAMPs activating C-type lectin receptors and NOD-like receptors on SCI-NHO. C57BL/6 mice underwent SCI plus muscle injury via an intramuscular injection of CDTX (0.3125 mg/kg). **A** NHO bone volumes at days 7 and 21 in mice treated post-surgery with vehicle or zymosan (15 and 30 mg/kg i.p.) with representative μCT images at 7 days post-surgery. **B** NHO bone volumes at 7 days post-surgery in mice treated post-surgery with vehicle or furfurman (5 and 20 mg/kg i.p.), with representative μCT images at 7 days post-surgery. **C** NHO bone volumes at days 7 and 21 in mice treated post-surgery with vehicle or GlcC14C18 (4 and 10 mg/kg i.p.) 24 h post-surgery with representative μCT images at 7 days post-surgery. **D** NHO bone volumes at days 7 and 21 in mice treated post-surgery with vehicle or Tri-DAP (1 and 2 mg/kg i.p.) post-surgery with representative μCT images at 7 days post-surgery Each dot represents an individual mouse, data represented as mean ± SD, one-way ANOVA. *p < 0.05, **p < 0.01, ***p < 0.001, ****p < 0.0001

The effects of C-type lectin receptor agonists that mimic PAMPs produced by yeast species and mycobacteria were also tested in vivo. These included zymosan (Fig. 4A), a particulate β-glycan extract of *Saccharomyces cerevisiae* yeast cell wall that activates TLR2/TLR6 heterodimer and both Dectin-1 and Dectin-2 [45], furfurman (Fig. 4B), a particulate cell wall preparation from the human skin yeast *Malassezia furfur* which activates Dectin-2 [46], and the di-acylated glucose GlcC14C18 (Fig. 4C) which mimics glycolipids of mycobacteria cell walls and activates Mincle [47]. C-type lectin receptor agonists were administered intraperitoneally 24 h post-surgery. Both zymosan and GlcC14C18 exacerbated SCI-NHO development (Fig. 4A, C) whereas furfurman had no effect (Fig. 4B). This suggests that Mincle activation exacerbates NHO development in mice whereas Dectin-2 does not.

Finally, we tested the role of NOD-like receptors which sense bacterial peptidoglycans using the synthetic peptidoglycan M-Tri-DAP which activates intracellular NOD1 and to a lesser extent NOD2 [48]. M-Tri-DAP (Fig. 4D) had no effect on SCI-NHO development suggesting that the activation of NOD-like receptors does not alter NHO development in mice.

### Effect of PAMPs on human and mouse FAPs mineralization potential in vitro

**Fig. 5:**
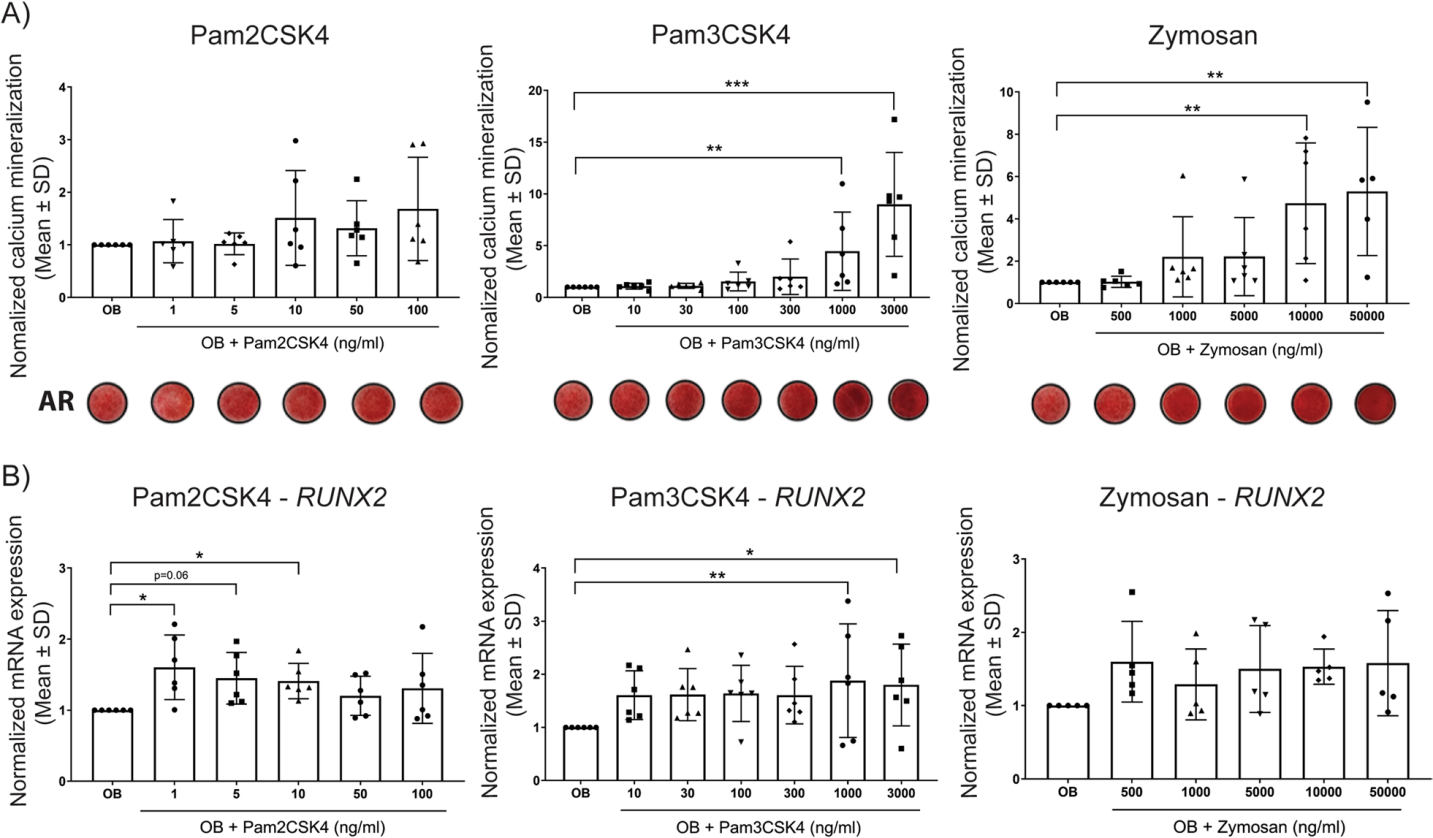
Direct effect of PAMPs on hFAPs mineralization. hFAPs (n = 6) were cultured for 2 weeks in osteogenic medium (OB) supplemented with PAMPs: Pam2CSK4 from 1 to 100 ng/ml; Pam3CSK4 from 10 to 3000 ng/ml; and Zymosan from 500 to 50,000 ng/ml. **A** Mineralization was visualized using Alizarin Red staining (AR) and quantified by spectrophotometry. **B** mRNA expression of the osteoblast marker *RUNX2* assessed by qRT-PCR. Each dot represents an individual donor. Data are represented as mean ± SD, one way ANOVA, *p < 0.05, **p < 0.01, ***p < 0.001

As the effects of PAMPs could not be tested in humans in vivo, we used surrogate in vitro assays by investigating the direct effect of PAMPs on PDGFRα^+^ hFAPs in vitro. PDGFRα^+^ CD56^−^ hFAPs were sorted from muscles surrounding NHO biopsies and cultured under osteogenic conditions with increasing concentrations of different PAMPs for 2 weeks, and mineralization was measured by Alizarin Red deposition. Quantification of mineralization demonstrated a trend of increased mineralization in hFAPs cultured with Pam2CSK4 but this did not reach statistical significance (Fig. 5A), however increasing concentrations of Pam3CSK4 and Zymosan significantly enhanced hFAPs mineralization compared to the controls (Fig. 5A). hFAPs cultured with Pam3CSK4 also significantly increased the expression of *RUNX2* mRNA (Fig. 5B). From the other PAMPs tested, only furfurman at the highest dosage increased hFAP calcium mineralization, and all other PAMPs tested either had no effect (flagellin, CpG ODN, gardiquimod, M-Tri-DAP and TDB) or were toxic to these cultures (poly (I:C)), (Fig. S2).

We also examined the direct effect of PAMPs in CD45^–^Ter119^–^CD31^–^CD34^+^Sca1^+^ murine FAPs sorted from the hamstring muscles of naive wild-type C57BL/6 mice. Murine FAPs were cultured in osteogenic conditions in the presence of Pam2CSK4 and Zymosan (Fig. S3A), and a similar dose dependent increase in calcium mineralization was observed. PAMPs such as Pam3CSK4, PGN-SA, M-TriDAP, CpG ODN1668, Furfurman and GlCC_14_C_18_ had no direct effect on FAP mineralization, while PAMPs such as poly(I:C), flagellin and gardiquimod caused cell death in culture, and therefore reduced FAP mineralization (Fig. S4).

Overall, this suggests that some PAMPs can stimulate FAP mineralization directly in both human and mouse muscles albeit with heterogeneity between both species.

### Indirect effects of PAMPs on human and mouse FAPs mineralization via macrophages

**Fig. 6:**
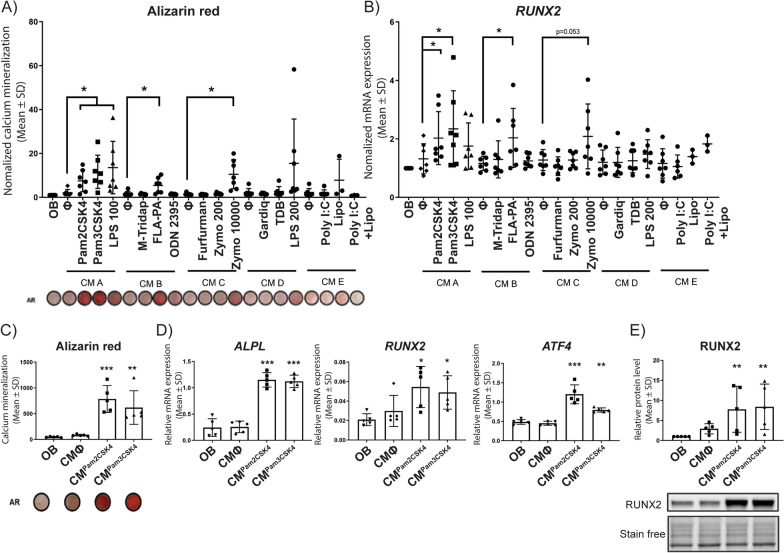
Indirect effect of PAMPs on hFAPs mineralization**.** CD14^+^ macrophages were isolated from 5 healthy donors and cultured with either no stimulation (∅), 200 ng/ml Pam2CSK4, 200 ng/ml Pam3CSK4, 100 ng/ml LPS (LPS 100), 200 ng/ml M-TriDAP, 200 ng/ml flagellin (FLA-PA), 200 ng/ml CpG ODN 2395, 200 ng/ml furfurman, 200 ng/ml Zymosan (Zymo 200), 10 µg/ml Zymosan (Zymo10 000), 200 ng/ml Gardiquimod (Gardiq), 200 ng/ml TDB, 200 ng/ml LPS (LPS 200), 200 ng/ml Poly(I:C), lipofectamine (Lipo) or 200 ng/ml Poly(I:C) + lipofectamine. These 5 donor monocyte-conditioned media samples (CM A-E) were added at 10% (v/v) to hFAPs isolated from between 3 and 7 NHO biopsies/patients for each condition and subsequently cultured for 2 weeks in osteogenic medium (OB) supplemented with conditioned medium (CM) from human CD14^+^ macrophages stimulated with indicated PAMPs. **A** Mineralization was visualized using Alizarin Red staining (AR) and quantified by spectrophotometry. **B** mRNA expression of the osteoblast marker *RUNX2* assessed by qRT-PCR. **C, D** FAPs (n = 5 donors) were cultured for 2 weeks in osteogenic medium (OB) supplemented with conditioned medium from human CD14^+^ macrophages stimulated with 200 ng/ml Pam2CSK4 (CM^Pam2CSK4^), 200 ng/ml Pam3CSK4 (CM^Pam3CSK4^) or non-stimulated (CM^∅^). **C** Mineralization was visualized using Alizarin Red staining (AR) and quantified by spectrophotometry. **D** mRNA expression of osteoblast markers *ALPL*, *RUNX2*, *ATF4* were assessed by qRT-PCR. **E** RUNX2 protein quantification by Western blot using Stain free normalization. Each dot represents an individual FAP donor. Data are represented as Mean ± SD, t-test, *p < 0.05 in (**A**) and (**B**); One-way ANOVA Dunnett’s multiple comparison test versus OB, *p < 0.05, **p < 0.01, ***p < 0.001 in (**C**–**E**)

As shown in Fig. 1, the majority of PRRs in humans are expressed by CD14^+^cells. Therefore, to test a potential indirect role of PAMPs influencing hFAP mineralization via monocyte/macrophages, we used conditioned media (CM) produced by human peripheral blood CD14^+^ cells from 5 healthy donors (A, B, C, D and E). CD14^+^ cells were cultured for 3 days with or without the indicated PAMPs (Fig. 6A). CM were added to hFAPs isolated from NHO patients and cultured in osteogenic medium, and mineralization was measured after 2 weeks. CM from CD14^+^ cells stimulated with Pam2CSK4, Pam3CSK4, flagellin, zymosan and LPS significantly increased hFAP mineralization when compared to CM from unstimulated CD14^+^ monocytes, whereas monocyte conditioning with other PAMPs (poly(I:C), gardiquimod, CpG ODN. M-TriDAP, furfurman and TDB) had no effect (Fig. 6A). Likewise, CM from CD14^+^ cells stimulated with Pam2CSK4, Pam3CSK4, flagellin and zymosan significantly enhanced *RUNX2* expression in hFAPs, whereas monocyte conditioning with other PAMPs had no effect on *RUNX2* expression (Fig. 6B). These data suggest that amongst all the PAMPs tested, Pam2CSK4, Pam3CSK4, flagellin, zymosan and LPS display the best potential to promote hFAPs mineralization and osteoblastic differentiation indirectly via monocytes/macrophages.

We next confirmed our results with additional donors (Fig. 6C) with the PAMPs demonstrating the most robust effects on hFAP mineralization. Using the same protocol, CM from human CD14^+^ monocytes unstimulated or stimulated with the TLR1/TLR2 ligand Pam2CSK4 or the TLR2/TLR6 ligand Pam3CSK4 were collected from 9 additional donors. CM from CD14^+^ monocytes stimulated with either Pam2CSK4 or Pam3CSK4 significantly promoted hFAPs mineralization (Fig. 6C). Using these additional donors, we then demonstrated that hFAPs cultured in Pam2CSK4 and Pam3CSK4 CM resulted in an increase in *ALPL*, *RUNX2* and *ATF4* mRNA expression (Fig. 6D) and increased RUNX2 protein (Fig. 6E), suggesting hFAPs osteoblastic differentiation.

Similar to human cells, we also found that CM from PAMP-stimulated mouse bone marrow derived macrophages significantly enhanced calcium mineralization of mouse muscle FAPs in vitro (Fig. S3B). Overall, these data show that several PAMPs can indirectly enhance the mineralization potential of muscle FAPs via monocyte/macrophages in humans and mice such as the TLR4 ligand LPS and the TLR2 ligands Pam2CSK4, Pam3CSK4 and zymosan.

### TLR2 inhibition reduces NHO bone development after administration of TLR1/2 agonist Pam3CSK4

As we observed that TLR2 ligands promoted both NHO in mice (Fig. 2A, B) and enhanced osteoblastic differentiation of human FAPs (Figs. 5 and 6), we tested the feasibility of TLR2 inhibition as a potential therapeutic intervention in our NHO mouse model. Similar to experiments outlined above, all mice underwent SCI and a CDTX-induced hamstring muscle injury. Mice were administered a small synthetic TLR2 inhibitor C29 (30 mg/kg) [49] immediately following surgery and 20 min before the administration of the TLR1/2 ligand Pam3CSK4 (8 mg/kg ip), with C29 daily treatment until day 3 post-surgery. NHO bone volumes at 7 days post-surgery established that TLR2 blockade significantly reduced NHO bone volumes in response to Pam3CSK4 compared to vehicle treated controls (Fig S5).

### Role of OSM and IL-1 in PAMP stimulation

We have previously shown that the inflammatory cytokines IL-1α, IL-1β and OSM are important drivers of NHO pathogenesis in mice and humans [15, 16, 34]. Thus, we investigated whether these cytokines are involved in the stimulatory effect of PAMPs on NHO development in mice and osteogenic differentiation of human FAPs.

Firstly, we measured OSM and IL-1β mRNA expression in injured muscles of mice that underwent SCI and muscle injury and then further challenged with zymosan, Pam2CSK4 or Pam3CSK4. Zymosan and Pam2CSK4 increased *Osm* but not *Il1b* mRNA expression, whereas Pam3CSK4 increased *Il1b* but not *Osm* mRNA expression in injured muscle (Fig.S6).

**Fig. 7:**
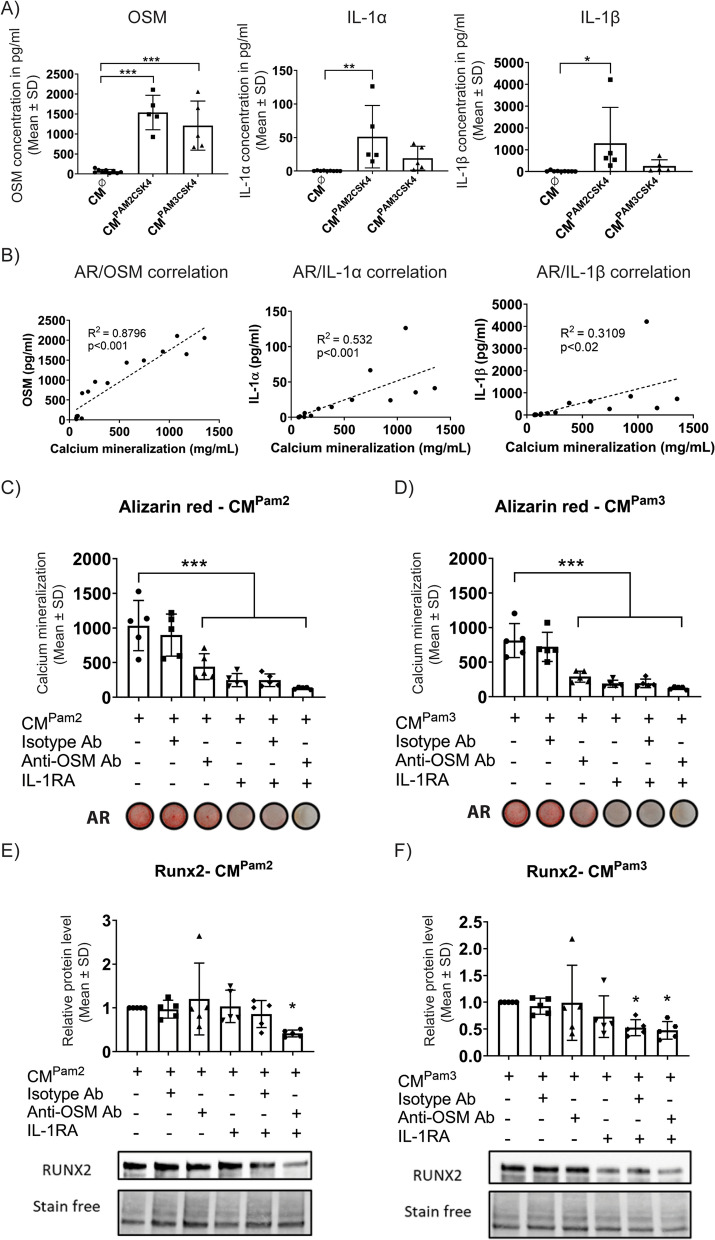
OSM and IL-1 neutralization in monocyte-conditioned media strongly inhibit hFAPs mineralization. **A** OSM, IL-1α and IL-1β concentrations quantified in conditioned media from human CD14^+^ macrophages stimulated with 200 ng/ml Pam2CSK4 (CM^Pam2CSK4^), 200 ng/ml Pam3CSK4 (CM^Pam3CSK4^) or non-stimulated (CM^∅^). **B** OSM, IL-1α and IL-1β concentrations in CM^Pam2CSK4^, CM^Pam3CSK4^ and CM^∅^ were correlated with hFAP calcium mineralization measured by Alizarin Red staining after 2 weeks of culture in osteogenic conditions in the presence of the conditioned media. Each dot represents a different conditioned medium sample. **C–F** Mouse anti-human OSM antibody, isotype control antibody and IL-1RA were used to neutralize OSM and IL-1 in human CD14^+^ monocyte conditioned media as indicated below each chart. Calcium mineralization of hFAPs cultured for 2 weeks in osteogenic conditions with **C** CM^Pam2CSK4^ or **D** CM^Pam3CSK4^ was measured using Alizarin Red staining and quantified by spectrophotometry. **E, F** RUNX2 protein quantification by Western blot using Stain-Free normalization for hFAPs cultured for 2 weeks in osteogenic conditions with **E** CM^Pam2CSK4^ or **F** CM^Pam3CSK4^. In C-F, each dot represents a hFAP individual donor. Bars represent mean ± SD, One-way ANOVA Dunnett’s multiple comparison test versus CM^∅^ in (**A**) and (**B**), versus CM^Pam2CSK4^ in (**C**) and (**E**), or versus CM^Pam3CSK4^ in (**D**) and (**F**), *p < 0.05, **p < 0.01, ***p < 0.001

In respect to human cells, we quantified OSM, IL-1α, and IL-1β proteins in CM from human CD14^+^ cells stimulated with Pam2CSK4 or Pam3CSK4. Secretion of OSM was robustly induced by Pam2CSK4 and Pam3CSK4 stimulation (Fig. 7A) whereas IL-1α and IL-1β secretion were significantly induced by Pam2CSK4 only (Fig. 7A). We also found significant positive correlations between OSM, IL-1α, and IL-1β protein concentrations in CM and hFAPs mineralization, with OSM showing the strongest correlation (R^2^ = 0.8796) (Fig. 7B).

To confirm the role of OSM, IL-1α and IL-1β secreted by PAMP-stimulated CD14^+^ cells in promoting hFAPs mineralization, we next used an inhibition strategy. hFAPs were cultured in osteogenic medium with Pam2CSK4 or Pam3CSK4-stimulated CD14^+^ cells CM and neutralizing anti-OSM antibody and/or IL-1 receptor antagonist (IL-1RA) were added to the culture medium. After 2 weeks, we observed a significant reduction in hFAPs mineralization activity using either anti-OSM antibody, IL-1RA or the combination of both (Fig. 7C, D). RUNX2 protein expression was also significantly reduced when OSM and IL-1 were inhibited (Fig. 7E, F). These results suggest that inflammatory cytokines secreted by PAMP-stimulated CD14^+^ monocytes can enhance hFAP mineralization and further highlight the key role of OSM and IL-1 signaling pathways in NHO pathophysiology.

## Discussion

Retrospective studies in patients with SCI and TBI have previously shown a significant association between the incidence of NHO and accompanying local and systemic infections [21, 50–53]. Recently, we demonstrated a functional association between gram-negative bacterial infections particularly with *Pseudomonas aeruginosa* and NHO development, where the PAMP LPS exacerbated NHO development in mice in a TLR4‐dependent manner and signaling through the TRIF/TICAM1 adaptor [32]. We now expand on this work and demonstrate that this phenomenon is not limited to LPS:TLR4 signaling, as PAMPs that target other PRRs also exacerbate NHO development in mice and increase calcium mineralization of human muscle FAPs in vitro.

Indeed fungal, viral and additional bacterial PAMPs exacerbated NHO bone volumes in mice in vivo, and the majority of these PAMPs significantly increased calcium mineralization of mouse FAPs, the NHO cells-of-origin [15], indirectly via macrophages. However, comparisons between mouse and human are more complex with varying effects of each PAMP (summarized in Table S4). Interestingly, we saw similar observations when using the lipopeptides Pam2CSK4 and Pam3CSK4 and the β-glucan zymosan. These PAMPs exacerbated NHO development in mice after SCI and also enhanced muscle FAP calcium mineralization directly or indirectly via monocyte/macrophages in both humans and mice. Both mouse and human monocytes expressed the receptors for these PAMPs namely TLR1, TLR2, TLR6 (on plasma membrane) and Dectin-1/Dectin-2 (cytosolic). The other PAMPs tested had heterogenous effects in mouse versus human which likely reflects the differences between mouse and human PRR expression patterns, and functional immune responses downstream of these PRRs [54, 55].

We previously demonstrated that LPS was able to enhance calcium mineralization of human FAPs in vitro directly [32], or indirectly via conditioned medium from macrophages stimulated with LPS [16, 56]. Therefore, we examined if additional PAMPs had similar potential using both mouse and human in vitro FAP cultures. In murine FAP cultures, only Pam2CSK4 and Zymosan administration resulted in increased calcium mineralization, whereas all other PAMPs tested had insignificant or toxic effects. This is most likely due to the lack of expression for the corresponding PRR on murine FAPs, with the exception of Poly (I:C), where we find murine FAPs express both the endosomal and cytosolic receptors for this PAMP. However, Poly(I:C), flagellin, and gardiquimod all reduced mouse FAP cell viability. Similar toxic effects have been previously observed with LPS on bovine mammary epithelial cells and Poly(I:C) on murine intestinal crypt cells [57, 58]. In hFAP cultures, only Pam3CSK4, zymosan and furfurman were able to directly enhance calcium mineralization and qRT-PCR confirmed hFAPs express PRRs for these PAMPs (albeit at low levels). The lack of expression of several PRRs in human FAPs likely explains why direct incubation with CpG oligodeoxynucleotide (TLR9 ligand) or Gardiquimod (TLR7 and TLR8 ligand) did not stimulate human FAP mineralization.

In contrast, all PAMPs tested were able to indirectly stimulate mouse FAP mineralization via macrophages. As expected, mouse muscle macrophages expressed all PRRs, albeit at varying levels. We were unable to isolate sufficient numbers of macrophages from injured mouse muscles to perform PAMP in vitro simulations to collect conditioned media, therefore for these in vitro assays we used mouse bone marrow-derived macrophages. For human in vitro studies, indirect stimulation of hFAPs using conditioned media from Pam2CSK4-, Pam3CSK4-, Zymosan- and furfurman-stimulated CD14^+^ monocytes increased calcium mineralization, and as expected, we found a wide range of PRRs expressed by human monocytes.

We have previously demonstrated that the pro-inflammatory cytokines IL-1 and OSM, which are secreted by activated monocytes/macrophages, are pivotal in NHO development in both humans and mice [15, 16]. We now report that OSM, IL-1α and IL-1β were secreted by human blood CD14^+^ monocytes stimulated with Pam2CSK4 or Pam3CSK4 and antibody-mediated neutralization of these cytokines significantly reduced human FAP mineralization in response to these two PAMPs in vitro. Pam2CSK4 and Pam3CSK4 also strongly enhanced NHO development in response to SCI in mice as well as *Osm* and *Il1b* mRNA expression in injured muscles developing NHO. Further to this, we found that treatment with a small synthetic TLR2 antagonist reduced NHO development in response to Pam3CSK4 in mice. Together these data suggest that these inflammatory cytokines mediate the increase in hFAP mineralization and NHO in mice in response to Pam2CSK4 and Pam2CSK4 via TLR2, similar to the induction of these cytokines in response to LPS via TLR4 that we previously demonstrated [16, 32]. From these observations, it is reasonable to speculate that the enhancing effects of other PAMPs on NHO described herein, may also be mediated by enhanced secretion of IL-1 and OSM by activated macrophages.

In addition, we have recently demonstrated that NHO development is driven by endogenous glucocorticoids released in response to the SCI. Interestingly, the administration of the glucocorticoid receptor agonist dexamethasone promoted heterotopic ossification development without SCI, and upregulated the expression of OSM and IL-1β as well as their cognate receptors OSMR and IL1R1 [12]. All of which highlights the importance of these two pro-inflammatory pathways during exposure to PAMPs and NHO development after SCI. Importantly, PAMPs such as LPS are already known to induce endogenous glucocorticoid release in humans [59] and mice [60]. Whether endogenous glucocorticoid secretion as a consequence of PAMP administration contributes to the enhancing effect of PAMPs to NHO development is a current line of investigation.

While our study focused on the role of PAMPs exacerbating NHO, it is important to acknowledge that PRRs also recognize DAMPs (such as HMBG1, hyaluronan, S100 family proteins), which are released from cells or extracellular matrix after tissue damage and elicit similar downstream immune signalling pathways to that of PAMPs [61–63]. In our NHO mouse model, the formation of NHO requires both a SCI and muscle damage [14], however the combination of SCI and LPS (without muscle damage) was not sufficient to elicit NHO, potentially highlighting a role for DAMPs in initiating NHO development.

Finally, a limitation of this study was that we were not able to administer live pathogens to our mice after surgery. The administration of live pathogens has the potential to elicit different responses to those seen in this study with purified PAMPS. In addition, all human results in this study are with in vitro cultures due to the ethical limitations. While our previous study [32] identified gram-negative infections as a risk factor for NHO development, our study did not have enough power to investigate contributions from gram-positive versus gram-negative, nor contributions from viral or fungal pathogens. Future prospective studies to identify a link between different types of infection with NHO incidence in larger cohorts of patients with neurological injury are warranted.

## Conclusions

Overall, this study further highlights the importance of infection management in patients with neurological injury. Importantly, our study now highlights that fungal PAMPs can exacerbate NHO development in mice. As several retrospective studies have identified urinary tract infections as a significant risk factor of NHO in SCI and TBI patients [18, 21, 22, 32] and catheters have been shown to support colonization and biofilm formation by *Candida* species, broad microbial vigilance, adapted prophylaxis and potentially TLR antagonist therapy will be needed for these patients to reduce the incidence of NHO.

## Supplementary Information

Additional file 1.

## Data Availability

Data will be available from the corresponding author upon request.

## References

[1] Garland DE. Clinical observations on fractures and heterotopic ossification in the spinal cord and traumatic brain injured populations. Clin Orthop Relat Res. 1988;233:86–101. 3135969

[2] Genet F, Jourdan C, Schnitzler A, Lautridou C, Guillemot D, Judet T, et al. Troublesome heterotopic ossification after central nervous system damage: a survey of 570 surgeries. PLoS ONE. 2011;6(1):e16632. 21304993 10.1371/journal.pone.0016632 PMC3031592

[3] Genêt F, Minooee K, Jourdan C, Ruet A, Denormandie P, Schnitzler A. Troublesome heterotopic ossification and stroke: features and risk factors. A case control study. Brain Inj. 2018;29(7–8):866–71. 10.3109/02699052.2015.1005133 25915823

[4] Alexander KA, Tseng H-W, Salga M, Genêt F, Levesque J-P. When the nervous system turns skeletal muscles into bones: how to solve the conundrum of neurogenic heterotopic ossification. Curr Osteoporos Rep. 2020;18(6):666–76. 33085000 10.1007/s11914-020-00636-w

[5] Genet F, Chehensse C, Jourdan C, Lautridou C, Denormandie P, Schnitzler A. Impact of the operative delay and the degree of neurologic sequelae on recurrence of excised heterotopic ossification in patients with traumatic brain injury. J Head Trauma Rehabil. 2012;27(6):443–8. 22495100 10.1097/HTR.0b013e31822b54ba

[6] Genet F, Jourdan C, Lautridou C, Chehensse C, Minooee K, Denormandie P, et al. The impact of preoperative hip heterotopic ossification extent on recurrence in patients with head and spinal cord injury: a case control study. PLoS ONE. 2011;6(8):e23129. 21853078 10.1371/journal.pone.0023129 PMC3154269

[7] Genet F, Marmorat JL, Lautridou C, Schnitzler A, Mailhan L, Denormandie P. Impact of late surgical intervention on heterotopic ossification of the hip after traumatic neurological injury. J Bone Jt Surg Br. 2009;91(11):1493–8. 10.1302/0301-620X.91B11.22305 19880896

[8] Salga M, Jourdan C, Durand MC, Hangard C, Denormandie P, Carlier RY, et al. Sciatic nerve compression by neurogenic heterotopic ossification: use of CT to determine surgical indications. Skeletal Radiol. 2015;44(2):233–40. 25218150 10.1007/s00256-014-2003-6

[9] de l’Escalopier N, Salga M, Gatin L, Genêt F, Denormandie P. Resection of heterotopic ossification around the hip after trauma. EFORT Open Rev. 2019;4(6):263–8. 31210967 10.1302/2058-5241.4.180098 PMC6549106

[10] Almangour W, Schnitzler A, Salga M, Debaud C, Denormandie P, Genêt F. Recurrence of heterotopic ossification after removal in patients with traumatic brain injury: a systematic review. Ann Phys Rehabil Med. 2016;59(4):263–9. 27173174 10.1016/j.rehab.2016.03.009

[11] Zakrasek EC, Yurkiewicz SM, Dirlikov B, Pence BT, Crew JD. Use of nonsteroidal anti-inflammatory drugs to prevent heterotopic ossification after spinal cord injury: a retrospective chart review. Spinal Cord. 2019;57(3):214–20. 30254206 10.1038/s41393-018-0199-3

[12] Alexander KA, Tseng HW, Lao HW, Girard D, Ungerer JPJ, McWhinney BC, et al. A glucocorticoid spike derails muscle repair to heterotopic ossification after spinal cord injury. Cell Rep Med. 2024;5:101849. 39657663 10.1016/j.xcrm.2024.101849 PMC11722129

[13] Debaud C, Tseng H-W, Chedik M, Kulina I, Genêt F, Ruitenberg M, et al. Local and systemic factors drive ectopic osteogenesis in regenerating muscles of spinal cord-injured mice in a lesion level-dependent manner. J Neurotrauma. 2021;38(15):2162–75. 33913747 10.1089/neu.2021.0058

[14] Genêt F, Kulina I, Vaquette C, Torossian F, Millard S, Pettit AR, et al. Neurological heterotopic ossification following spinal cord injury is triggered by macrophage-mediated inflammation in muscle. J Pathol. 2015;236(2):229–40. 25712044 10.1002/path.4519

[15] Tseng H-W, Kulina I, Girard D, Gueguen J, Vaquette C, Salga M, et al. Interleukin-1 is overexpressed in injured muscles following spinal cord injury and promotes neurogenic heterotopic ossification. J Bone Miner Res. 2022;37(3):531–46. 34841579 10.1002/jbmr.4482

[16] Torossian F, Guerton B, Anginot A, Alexander KA, Desterke C, Soave S, et al. Macrophage-derived oncostatin M contributes to human and mouse neurogenic heterotopic ossifications. JCI Insight. 2017;2(21):e96034. 29093266 10.1172/jci.insight.96034 PMC5752299

[17] Tseng H-W, Girard D, Alexander KA, Millard SM, Torossian F, Anginot A, et al. Spinal cord injury reprograms muscle fibroadipogenic progenitors to form heterotopic bones within muscles. Bone Res. 2022;10(1):22. 35217633 10.1038/s41413-022-00188-y PMC8881504

[18] Citak M, Suero EM, Backhaus M, Aach M, Godry H, Meindl R, et al. Risk factors for heterotopic ossification in patients with spinal cord injury: a case-control study of 264 patients. Spine (Phila Pa 1976). 2012;37(23):1953–7. 22614800 10.1097/BRS.0b013e31825ee81b

[19] Dizdar D, Tiftik T, Kara M, Tunc H, Ersoz M, Akkus S. Risk factors for developing heterotopic ossification in patients with traumatic brain injury. Brain Inj. 2013;27(7–8):807–11. 23730889 10.3109/02699052.2013.775490

[20] Hendricks HT, Geurts AC, van Ginneken BC, Heeren AJ, Vos PE. Brain injury severity and autonomic dysregulation accurately predict heterotopic ossification in patients with traumatic brain injury. Clin Rehabil. 2007;21(6):545–53. 17613585 10.1177/0269215507075260

[21] Reznik JE, Biros E, Marshall R, Jelbart M, Milanese S, Gordon S, et al. Prevalence and risk-factors of neurogenic heterotopic ossification in traumatic spinal cord and traumatic brain injured patients admitted to specialised units in Australia. J Musculoskelet Neuronal Interact. 2014;14(1):19–28. 24583537

[22] Suero EM, Meindl R, Schildhauer TA, Citak M. Clinical prediction rule for heterotopic ossification of the hip in patients with spinal cord injury. Spine (Phila Pa 1976). 2018;43(22):1572–8. 29652785 10.1097/BRS.0000000000002680

[23] van Kampen PJ, Martina JD, Vos PE, Hoedemaekers CW, Hendricks HT. Potential risk factors for developing heterotopic ossification in patients with severe traumatic brain injury. J Head Trauma Rehabil. 2011;26(5):384–91. 21321512 10.1097/HTR.0b013e3181f78a59

[24] Mezghani S, Salga M, Tordjman M, Amar R, Carlier R-Y, Chiche L. Heterotopic ossification and COVID 19: imaging analysis of ten consecutive cases. Eur J Radiol. 2022;152:110336. 35523038 10.1016/j.ejrad.2022.110336 PMC9055793

[25] Stoira E, Elzi L, Puligheddu C, Garibaldi R, Voinea C, Chiesa AF, et al. High prevalence of heterotopic ossification in critically ill patients with severe COVID-19. Clin Microbiol Infect. 2021;27(7):1049–50. 33460831 10.1016/j.cmi.2020.12.037 PMC7833636

[26] Chaitani H, Fabeck L, Koulischer S. Heterotopic ossification following COVID-19 infections: systematic literature review of case reports and case series. BMC Musculoskelet Disord. 2024;25(1):421. 38811925 10.1186/s12891-024-07537-4 PMC11134613

[27] Chen GY, Nuñez G. Sterile inflammation: sensing and reacting to damage. Nat Rev Immunol. 2010;10(12):826–37. 21088683 10.1038/nri2873 PMC3114424

[28] Kumar H, Kawai T, Akira S. Pathogen recognition by the innate immune system. Int Rev Immunol. 2011;30(1):16–34. 21235323 10.3109/08830185.2010.529976

[29] Brubaker SW, Bonham KS, Zanoni I, Kagan JC. Innate immune pattern recognition: a cell biological perspective. Annu Rev Immunol. 2015;33:257–90. 25581309 10.1146/annurev-immunol-032414-112240 PMC5146691

[30] Oth T, Vanderlocht J, Van Elssen CH, Bos GM, Germeraad WT. Pathogen-associated molecular patterns induced crosstalk between dendritic cells, T helper cells, and natural killer helper cells can improve dendritic cell vaccination. Mediators Inflamm. 2016;2016:5740373. 26980946 10.1155/2016/5740373 PMC4766350

[31] Li D, Wu M. Pattern recognition receptors in health and diseases. Signal Transduct Target Ther. 2021;6(1):291. 34344870 10.1038/s41392-021-00687-0 PMC8333067

[32] Salga M, Samuel SG, Tseng HW, Gatin L, Girard D, Rival B, et al. Bacterial lipopolysaccharides exacerbate neurogenic heterotopic ossification development. J Bone Miner Res. 2023;38(11):1700–17. 37602772 10.1002/jbmr.4905

[33] Lilley E, Andrews MR, Bradbury EJ, Elliott H, Hawkins P, Ichiyama RM, et al. Refining rodent models of spinal cord injury. Exp Neurol. 2020;328:113273. 32142803 10.1016/j.expneurol.2020.113273

[34] Alexander KA, Tseng H-W, Fleming W, Jose B, Salga M, Kulina I, et al. Inhibition of JAK1/2 tyrosine kinases reduces neurogenic heterotopic ossification after spinal cord injury. Front Immunol. 2019;10:377. 30899259 10.3389/fimmu.2019.00377 PMC6417366

[35] Funderburg NT, Jadlowsky JK, Lederman MM, Feng Z, Weinberg A, Sieg SF. The Toll-like receptor 1/2 agonists Pam(3) CSK(4) and human β-defensin-3 differentially induce interleukin-10 and nuclear factor-κB signalling patterns in human monocytes. Immunology. 2011;134(2):151–60. 21896010 10.1111/j.1365-2567.2011.03475.x PMC3194223

[36] Kang JY, Nan X, Jin MS, Youn SJ, Ryu YH, Mah S, et al. Recognition of lipopeptide patterns by Toll-like receptor 2-Toll-like receptor 6 heterodimer. Immunity. 2009;31(6):873–84. 19931471 10.1016/j.immuni.2009.09.018

[37] Kang S-S, Sim J-R, Yun C-H, Han SH. Lipoteichoic acids as a major virulence factor causing inflammatory responses via Toll-like receptor 2. Arch Pharm Res. 2016;39(11):1519–29. 27498542 10.1007/s12272-016-0804-y

[38] Sester DP, Brion K, Trieu A, Goodridge HS, Roberts TL, Dunn J, et al. CpG DNA activates survival in murine macrophages through TLR9 and the phosphatidylinositol 3-kinase-Akt pathway. J Immunol. 2006;177(7):4473–80. 16982883 10.4049/jimmunol.177.7.4473

[39] Mizel SB, Honko AN, Moors MA, Smith PS, West AP. Induction of macrophage nitric oxide production by Gram-negative flagellin involves signaling via heteromeric Toll-like receptor 5/Toll-like receptor 4 complexes. J Immunol. 2003;170(12):6217–23. 12794153 10.4049/jimmunol.170.12.6217

[40] Zhao Y, Yang J, Shi J, Gong YN, Lu Q, Xu H, et al. The NLRC4 inflammasome receptors for bacterial flagellin and type III secretion apparatus. Nature. 2011;477(7366):596–600. 21918512 10.1038/nature10510

[41] Ma F, Zhang J, Zhang J, Zhang C. The TLR7 agonists imiquimod and gardiquimod improve DC-based immunotherapy for melanoma in mice. Cell Mol Immunol. 2010;7(5):381–8. 20543857 10.1038/cmi.2010.30 PMC4002679

[42] Alexopoulou L, Holt AC, Medzhitov R, Flavell RA. Recognition of double-stranded RNA and activation of NF-κB by Toll-like receptor 3. Nature. 2001;413(6857):732–8. 11607032 10.1038/35099560

[43] Kato H, Takeuchi O, Sato S, Yoneyama M, Yamamoto M, Matsui K, et al. Differential roles of MDA5 and RIG-I helicases in the recognition of RNA viruses. Nature. 2006;441(7089):101–5. 16625202 10.1038/nature04734

[44] Cassady KA, Gross M, Roizman B. The herpes simplex virus US11 protein effectively compensates for the gamma1(34.5) gene if present before activation of protein kinase R by precluding its phosphorylation and that of the alpha subunit of eukaryotic translation initiation factor 2. J Virol. 1998;72(11):8620–6. 9765401 10.1128/jvi.72.11.8620-8626.1998 PMC110273

[45] Gantner BN, Simmons RM, Canavera SJ, Akira S, Underhill DM. Collaborative induction of inflammatory responses by dectin-1 and toll-like receptor 2. J Exp Med. 2003;197(9):1107–17. 12719479 10.1084/jem.20021787 PMC2193968

[46] Ishikawa T, Itoh F, Yoshida S, Saijo S, Matsuzawa T, Gonoi T, et al. Identification of distinct ligands for the C-type lectin receptors Mincle and Dectin-2 in the pathogenic fungus Malassezia. Cell Host Microbe. 2013;13(4):477–88. 23601109 10.1016/j.chom.2013.03.008

[47] Decout A, Silva-Gomes S, Drocourt D, Barbe S, André I, Cueto FJ, et al. Rational design of adjuvants targeting the C-type lectin Mincle. Proc Natl Acad Sci USA. 2017;114(10):2675–80. 28223515 10.1073/pnas.1612421114 PMC5347620

[48] Girardin SE, Travassos LH, Hervé M, Blanot D, Boneca IG, Philpott DJ, et al. Peptidoglycan molecular requirements allowing detection by Nod1 and Nod2. J Biol Chem. 2003;278(43):41702–8. 12871942 10.1074/jbc.M307198200

[49] Guo T, Xiong L, Xie J, Zeng J, Huang Z, Yao M, et al. TLR2 promotes traumatic deep venous thrombosis of the lower extremity following femoral fracture by activating the NF-κB/COX-2 signaling pathway in rats. Exp Ther Med. 2024;28(6):436. 39355523 10.3892/etm.2024.12725 PMC11443593

[50] Lal S, Hamilton BB, Heinemann A, Betts HB. Risk factors for heterotopic ossification in spinal cord injury. Arch Phys Med Rehabil. 1989;70(5):387–90. 2497715

[51] Wittenberg RH, Peschke U, Botel U. Heterotopic ossification after spinal cord injury. Epidemiology and risk factors. J Bone Joint Surg Br. 1992;74(2):215–8. 1544955 10.1302/0301-620X.74B2.1544955

[52] Bravo-Payno P, Esclarin A, Arzoz T, Arroyo O, Labarta C. Incidence and risk factors in the appearance of heterotopic ossification in spinal cord injury. Paraplegia. 1992;30(10):740–5. 1448303 10.1038/sc.1992.142

[53] Cipriano C, Pill SG, Rosenstock J, Keenan MA. Radiation therapy for preventing recurrence of neurogenic heterotopic ossification. Orthopedics. 2009;32(9). 10.3928/01477447-20090728-33 19750999

[54] Mestas J, Hughes CC. Of mice and not men: differences between mouse and human immunology. J Immunol. 2004;172(5):2731–8. 14978070 10.4049/jimmunol.172.5.2731

[55] Ariffin JK, Sweet MJ. Differences in the repertoire, regulation and function of Toll-like receptors and inflammasome-forming Nod-like receptors between human and mouse. Curr Opin Microbiol. 2013;16(3):303–10. 23540353 10.1016/j.mib.2013.03.002

[56] Gueguen J, Girard D, Rival B, Fernandez J, Goriot ME, Banzet S. Spinal cord injury dysregulates fibro-adipogenic progenitors miRNAs signaling to promote neurogenic heterotopic ossifications. Commun Biol. 2023;6(1):932. 37700159 10.1038/s42003-023-05316-w PMC10497574

[57] Shi H, Guo Y, Liu Y, Shi B, Guo X, Jin L, et al. The in vitro effect of lipopolysaccharide on proliferation, inflammatory factors and antioxidant enzyme activity in bovine mammary epithelial cells. Anim Nutr. 2016;2(2):99–104. 29767022 10.1016/j.aninu.2016.03.005 PMC5941019

[58] Bou-Hanna C, Jarry A, Mosnier JF, Bossard C, Laboisse CL. The double stranded RNA analog poly-IC elicits both robust IFN-λ production and oncolytic activity in human gastrointestinal cancer cells. Oncotarget. 2018;9(77):34471–84. 30349642 10.18632/oncotarget.26121 PMC6195374

[59] Richardson RP, Rhyne CD, Fong Y, Hesse DG, Tracey KJ, Marano MA, et al. Peripheral blood leukocyte kinetics following in vivo lipopolysaccharide (LPS) administration to normal human subjects: Influence of elicited hormones and cytokines. Ann Surg. 1989;210(2):239–45. 2667476 10.1097/00000658-198908000-00018 PMC1357836

[60] Zuckerman SH, Shellhaas J, Butler LD. Differential regulation of lipopolysaccharide-induced interleukin 1 and tumor necrosis factor synthesis: effects of endogenous and exogenous glucocorticoids and the role of the pituitary-adrenal axis. Eur J Immunol. 1989;19(2):301–5. 2784766 10.1002/eji.1830190213

[61] Fink MP. Bench-to-bedside review: high-mobility group box 1 and critical illness. Crit Care. 2007;11(5):229. 17903310 10.1186/cc6088 PMC2556731

[62] Scheibner KA, Lutz MA, Boodoo S, Fenton MJ, Powell JD, Horton MR. Hyaluronan fragments act as an endogenous danger signal by engaging TLR2. J Immunol. 2006;177(2):1272–81. 16818787 10.4049/jimmunol.177.2.1272

[63] Roth J, Vogl T, Sorg C, Sunderkötter C. Phagocyte-specific S100 proteins: a novel group of proinflammatory molecules. Trends Immunol. 2003;24(4):155–8. 12697438 10.1016/s1471-4906(03)00062-0

