# Supplementary material for: Many but not all pathogen-associated molecular patterns aggravate neurogenic heterotopic ossification after spinal cord injury
Source: J Biomed Sci. 2026 Apr 10;33:39. doi: 10.1186/s12929-026-01237-y (PMC13067622; doi:10.1186/s12929-026-01237-y)
Supplement: Supplementary file 1 — Additional file 1. [file 12929_2026_1237_MOESM1_ESM.pdf]

Supplementary Tables and Figures

Many but not all pathogen-associated molecular patterns aggravate neurogenic heterotopic ossification after spinal cord injury

Selwin G Samuel[1,2], Hsu-Wen Tseng[1], Bastien Rival[3], Valerie Barbier[1], Kavita Bisht[1], Marjorie Salga[4,5], Shrutika Mate[1], Whitney Fleming[1], François Genêt[4,5], Sebastien Banzet[3], Jean-Pierre Lévesque[1*], Dorothée Girard[3*], Kylie A Alexander[1*]

Table S1 – Summary of PAMPs used in the study

PAMPs	Source	Cognate PRRs	Mode of injection	Vehicle
Pam3CSK4 lipopeptide	Invivogen Cat# tlrl-pms	TLR1/ TLR2	intraperitoneal	Saline
Pam2CSK4 lipopeptide	Invivogen Cat# tlrl-pm2s	TLR2/ TLR6	intraperitoneal	PBS
S. aureus Lipoteichoic acid	Invivogen Cat# tlrl-pslta	TLR2	intramuscular	PBS
S. aureus peptidoglycan	Invitrogen Cat#tlrl-pgns2	TLR2	In vitro only	Water
P. aeruginosa flagellin	Invivogen Cat# tlrl-pafla	TLR5	intraperitoneal	Saline
Unmethylated CpG phosphorothioate ODN 1668 5'-TCCATGACGTTCC TGATGCT-3'	Integrated DNA Technologies	Mouse TLR9	intramuscular	Control phosphorothioate ODN GC-1168 5'- TCCATGAGCTTCCT GATGC-3'
Unmethylated CpG phosphorothioate ODN 2395 5'-TCGTCGTTTTCGG CGCGCGCCG-3'	Invivogen Cat# tlrl-2395	Human TLR9	In vitro only with human cells	PBS
Gardiquimod	Invivogen Cat# tlrl-gdqs	TLR7	intraperitoneal	PBS
High molecular weight poly(I:C)	Invivogen Cat# tlrl-pic5	TLR3 (endosomal), MDA5, RIG-I and PKR (cytosolic)	intraperitoneal	PBS/Water
S. cerevisiae Zymosan	Invivogen Cat# tlrl-zyn	Dectin-1, Dectin-2 (cytosolic) and TLR2/6 (extracellular)	Intraperitoneal	PBS
Malassezia furfur Furfurman	Invivogen Cat# tlrl-ffm	Dectin-2	Intraperitoneal	PBS
Glucosyl – 6 – tetradecyl octadecenoate	Invivogen Cat# tlrl-gcc	Mincle	Intraperitoneal	DMSO (0.2%) in PBS
Trehalose-6,6-dibehenate (TDB)	Invivogen Cat# tlrl-tdb	Mincle	In vitro only with human cells	DMSO (0.2%) in PBS
M-TriDAP	Invivogen Cat# tlrl-mtd	NOD1 and NOD2	intraperitoneal	Saline

Table S2 – qRT-PCR primer probe sets for PRRs

Protein names	Mouse/Human Gene Symbols	Assay ID for mouse/human
TLR1	*Tlr1/TLR1*	Mm00446095_m1, Hs_TLR1_3_SG
TLR2	*Tlr2/TLR2*	Mm00442346_m1, Hs_TLR2_1_SG
TLR3	*Tlr3/TLR3*	Mm01207404_m1, Hs_TLR3_1_SG
TLR4	*Tlr4/TLR4*	Mm00445273_m1, Hs_TLR4_2_SG
TLR5	*Tlr5/TLR5*	Mm00546288_s1, Hs_TLR5_2_SG
TLR6	*Tlr6/TLR6*	Mm02529782_s1, Hs_TLR6_1_SG
TLR7	*Tlr7/TLR7*	Mm00446590_m1, Hs_TLR7_1_SG
TLR8	*Tr8//TLR8*	Hs_TLR8_2_SG
TLR9	*Tlr9/TLR9*	Mm00446193_m1, PPH01809A
Dectin-1	*Clec7a /CLEC7A*	Mm01183349_m1, Hs_CLEC7A_1_SG
Dectin-2	*Clec4n/CLEC6A*	Mm00490934_m1, Hs_CLEC6A_1_SG
NOD1	*Nod1/NOD1*	Mm00805062_m1, Hs_NOD1_1_SG
NOD2	*Nod2/NOD2*	Mm00467543_m1, Hs_NOD2_1_SG
PKR	*Prkar1a/PRKAR1A*	Mm01235643_m1, Hs_EIF2AK2_1_SG
RIG-I	*Rigi/RIGI*	Mm01216853_m1, Hs_DDX58_1_SG
MDA-5	*Ifih1/IFIH1*	Mm00459183_m1, Hs_IFIH1_1_SG
Mincle	*Clec4e/CLEC4E*	Mm01183703_m1, Hs_CLEC4E_1_SG
STING	*Sting1/STING1*	Mm01158117_m1, Hs_TMEM173_1_SG
Hypoxanthine guanine phosphoribosyl transferase	*Hprt/ HPRT*	Mm03024075_m1, Hs_HPRT1_1_SG
Ribosomal protein S20	*Rps20*	Mm02342828_g1
Oncostatin M	*Osm*	Mm01193966_m1
Interleukin-1β	*IL1β*	Mm00434228_m1
Actin Beta	*ACTB*	Hs_ACTB_1_SG

Peptidylprolyl isomerase A	*PPIA*	Hs_PPIA_1_SG
Ribosomal protein lateral stalk subunit P0	*RPLP0*	Hs_RPLP0_1_SG
Glyceraldehyde-3-phosphate dehydrogenase	*GAPDH*	Hs_GAPDH_1_SG
Alkaline phosphatase	*ALPL*	Hs_ALPL_1_SG
Activating transcription factor 4	*ATF4*	Hs_ATF4_1_SG
RUNX family transcription factor 2	*RUNX2*	Hs_RUNX2_1_SG

Table S3 – Summary of PRR expression in mouse and human muscle cells and leukocytes

PRRs	mSCs	mFAPs	mECs	mMΦ	hPDGFRa^{+}	hCD14^{+}
TLR1	-	-	-	(+)	Low	High
TLR2	-	(+++)	-	(+++)	Low	High
TLR3	(++)	(+++)	(+++)	(+)	High	-
TLR4	(+++)	(++)	(++)	(++)	Low	High
TLR5	-	(++)	-	(+)	Low	High
TLR6	-	(++)	-	(+)	Low	High
TLR7	-	-	-	(+)	-	High
TLR8	N/A	N/A	N/A	N/A	-	High
TLR9	-	-	-	(+)	-	High
STING	(++)	(+++)	(++)	(++)	High	-
RIG-1	(+)	(+)	(++)	(+)	High	High
MDA-5	(+++)	(+++)	(+++)	(++)	Low	High
PKR	(+++)	(+++)	(+++)	(++)	High	Low
NOD1	(++)	(++)	(+++)	(++)	High	Low
NOD2	-	(+)	(++)	(+)	-	High
Dectin-1	-	-	-	(++)	-	High
Dectin-2	(++)	-	-	(+++)	-	Low
Mincle	-	-	-	(+++)	-	High

mSCs mFAPs, mECs, mMΦ abbreviation represent respectively mouse satellite cells, fibro-adipogenic progenitors endothelial cells and macrophages sorted from hindlimb muscles. hPDGFRα+ and hCD14+ represent human PDGFRα+ FAPs sorted from NHO biopsies and CD14+ blood monocytes respectively . For mouse cells(+) represents expressed but less than splenocytes, (++) equivalent expression to splenocytes, (+++) expression higher than splenocytes, For human cells, expression of mRNA is summarized as low or high. – symbols represents not detected.

Table S4. Summary of in vivo and in vitro assay results PAMP effects on NHO development.

PAMPs	Mouse NHO volume	Direct effect on hFAP mineralization	Direct effect on hFAP Runx2 expression	Indirect effect on hFAP mineralization	Indirect effect on Runx2 expression
Pam3CSK4 lipopeptide	++	++ (1 and 3µg/ml)	++ (1 and 3 µg/ml)	++	++ (200ng/ml)
Pam2CSK4 lipopeptide	+++	+ (100ng/ml)	+ (1ng/ml)	++	+ (200ng/ml)
Poly (I:C)	+++	-	+ (3µg/ml)	-	-
Lipopolysaccharide	+++	+ (100ng/ml)	++ (10 and 100ng/ml)	+++	++ (100ng/ml)
Flagellin	-	-	+ (1 and 10 ng/ml)	++	+ (200ng/ml)
Gardiquimod	+++	-	+ (3µg/ml)	-	-
CpG phosphorothioate oligonucleotides	+++	-	-	-	-
Furfurman	-	+ (30µg/ml)	-	-	-
M-TriDAP (Muramyl tripeptide; peptidoglycan mimetic)	-	-	-	-	-
Mincle agonists Trehalose-6,6-dibehenate (TDB)	++	-	-	-	-
Zymosan (β-glucan)	+++	++ (10 and 50 µg/ml)	-	++	++ (10 µg/ml)

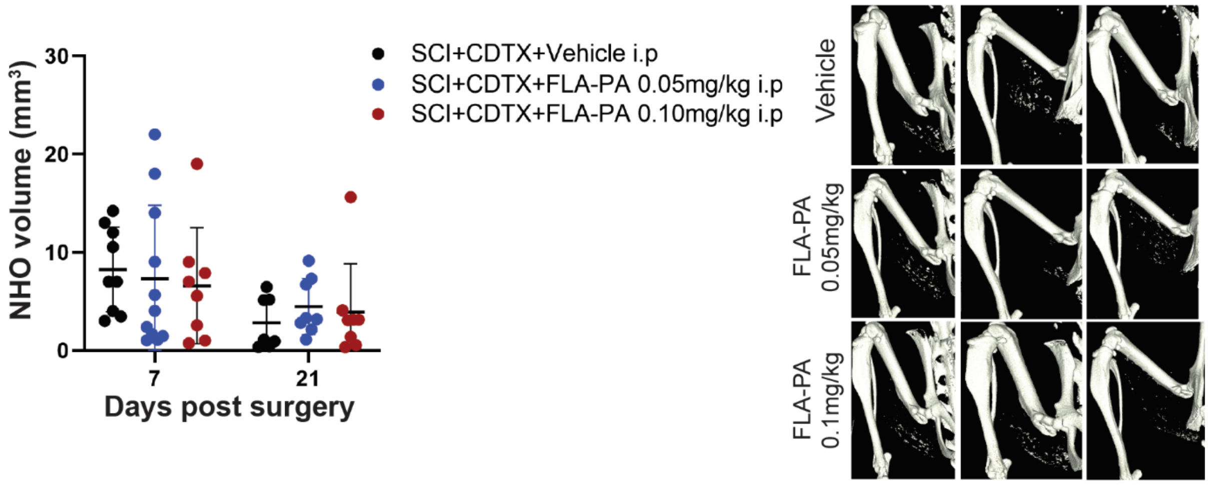

Fig. S1 Flagellin does not alter SCI-NHO development in mice. C57BL/6 mice underwent SCI plus muscle injury via an intramuscular injection of CDTX (0.3125mg/kg). NHO bone volumes at days 7 and 21 in mice treated post-surgery with vehicle or *P. aeruginosa* flagellin (FLA-PA) (0.05 or 0.1mg/kg), with representative μCT images at 7 days post-surgery. Each dot represents a separate mouse, data represented as mean ± SD, one-way ANOVA.

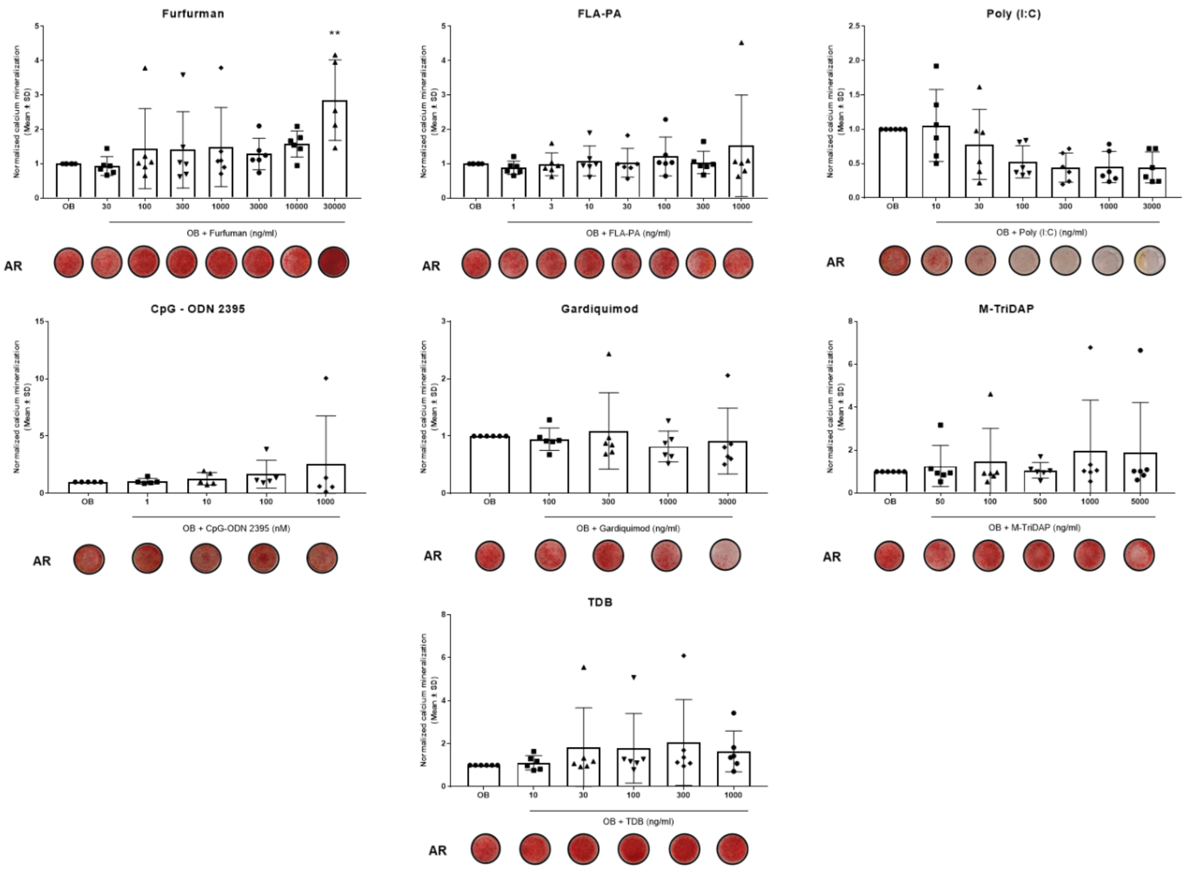

Fig S2 Direct effect of PAMPs on hFAPs mineralization. hFAPs (n=5,6) were cultured for 2 weeks in osteogenic medium (OB) supplemented with PAMPs: furfurman from 30 to 30000 ng/ml; FLA-PA (flagellin) from 1 to 1000 ng/ml; Poly(I:C) from 10 to 3000 ng/ml; CpG ODN from 1 to 1000 nM; Gardiquimod from 100 to 3000 ng/ml; M-TriDAP from 50 to 5000 ng/ml and TDB from 10 to 1000 ng/ml. Mineralization was visualized using Alizarin Red staining (AR) and quantified by spectrophotometry. Each dot represents an individual donor. Data are represented as mean ± SD, one way ANOVA, $**p<0.01$.

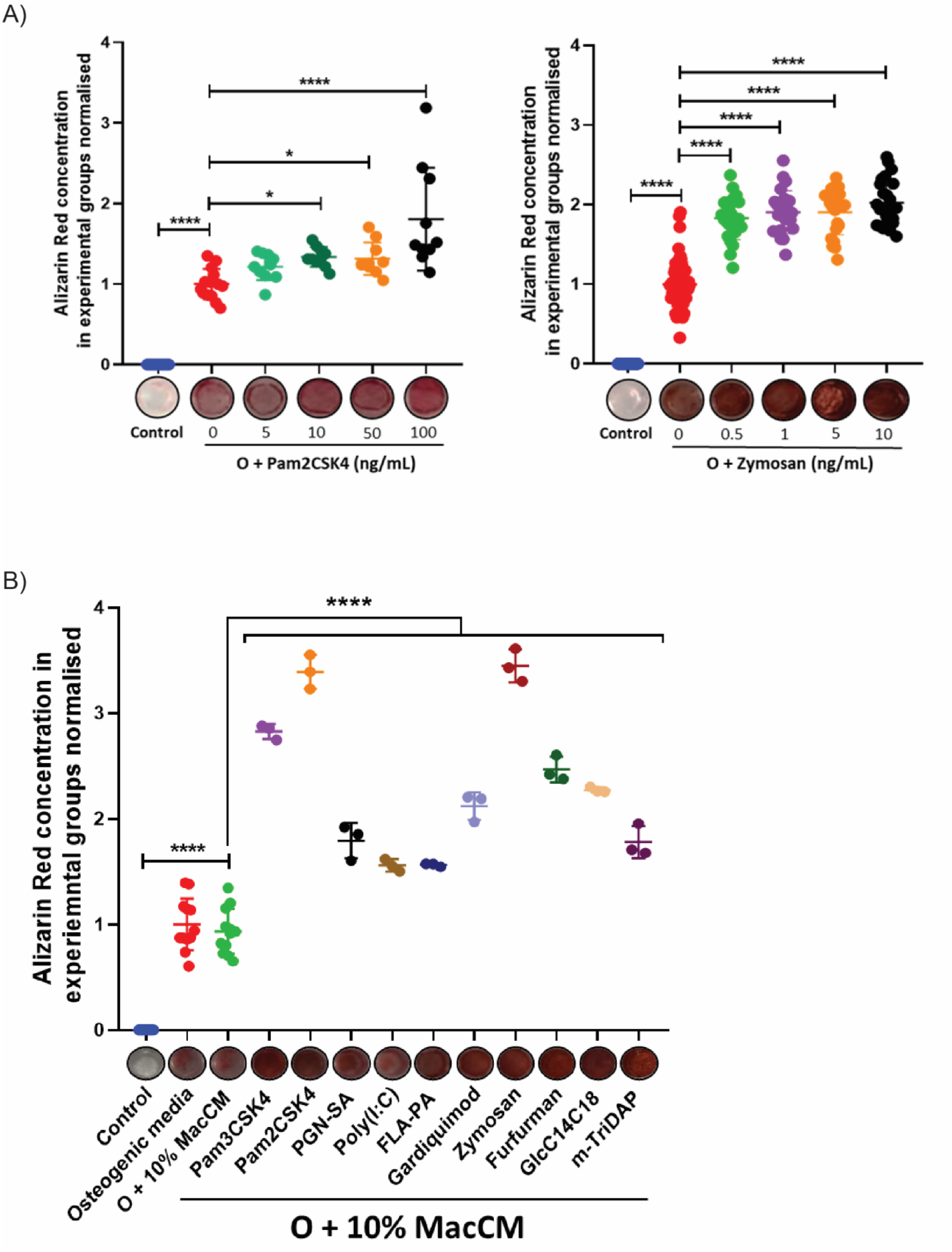

Fig S3 PAMPs directly and indirectly stimulate mouse FAP calcium mineralization. (A) Direct effect of zymosan or Pam2CSK4 stimulation on calcium mineralization of mouse muscle FAPs cultured in control or osteogenic medium (O). After 10-14 days in culture with the indicated concentrations of zymosan or Pam2CSK4, calcium mineralization was quantified using Alizarin Red staining. Data are represented as normalized absorbance values by

spectrophotometry at 562nm relative to osteogenic medium alone wells, across three individual experiments (average value = 1). (**B**) Indirect effect of PAMPs on mouse muscle FAP mineralization via macrophages. Mouse BMDMs were stimulated with indicated PAMPs for 18 hours and macrophage conditioned media (MacCM) were collected. 10% (v/v) MacCM were added to FAP cultures in osteogenic condition. Control MacCM is medium conditioned by macrophages in the absence of PAMP. One group of negative control wells contained cells cultured in a medium without the addition of osteogenic factors and conditioned media. All other wells were cultured in osteogenic conditions (O). Data are represented as normalized absorbance values at 562nm across four separate experiments. Absorbance values are relative to osteogenic medium alone wells (mean value = 1). For A) and B), each dot represents one well. Error bars represent mean ± SD. Statistical differences were calculated by ANOVA with post-hoc correction for multiple comparisons relative to osteogenic medium alone. * $p < 0.05$, **** $p < 0.0001$.

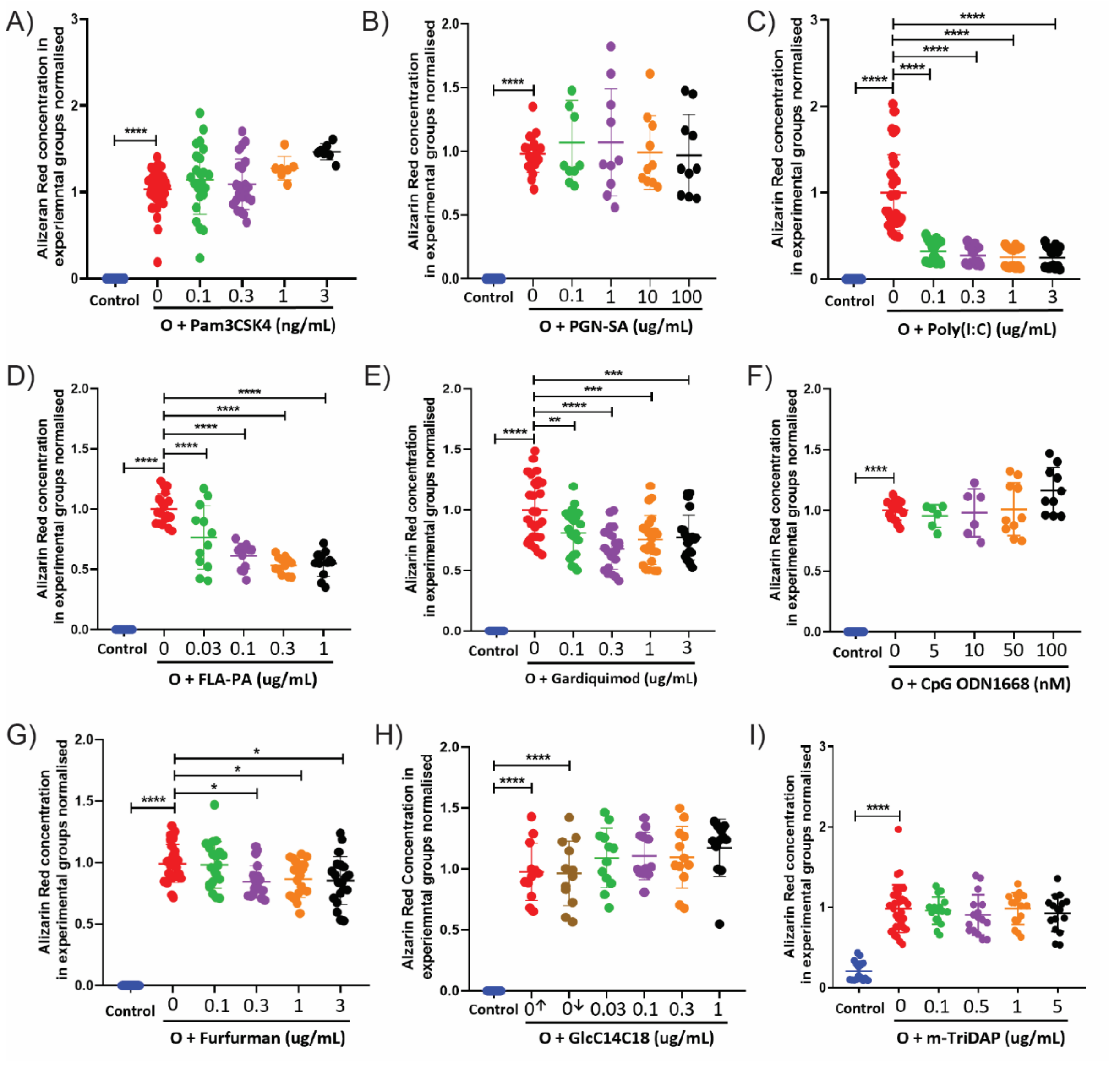

Fig S4 Direct effect of PAMPs on osteogenic mineralization potential of mouse FAPs. Sorted mouse muscle FAPs were cultured in control or osteogenic medium (O) with increasing concentrations of PAMPs: (**A**) Pam3CSK4, (**B**) peptidoglycan (PGN-SA), (**C**) Poly(I:C), (**D**) flagellin (FLA-PA), (**E**) Gardiquimod, (**F**) CpG ODN1668, (**G**) Furfurman, (**H**) GlCC$_{14}$C$_{18}$ and (**I**) M-TriDAP. For GlcC14C18, there were two positive controls, where ↑ and ↓ correspond to the concentrations of DMSO in wells treated with the highest dose (1µg/ml) and lowest dose (30ng/ml) of GlcC14C18. Data are represented as normalized absorbance values at 562nm relative to osteogenic medium alone wells (average value = 1), across two or three individual experiments. Each dot represents one well, data represented as mean ± SD. Statistical differences were calculated by one way ANOVA with post-hoc correction for multiple comparisons relative to osteogenic medium alone. * $p < 0.05$, ** $p < 0.01$, *** $p < 0.001$, **** $p < 0.0001$.

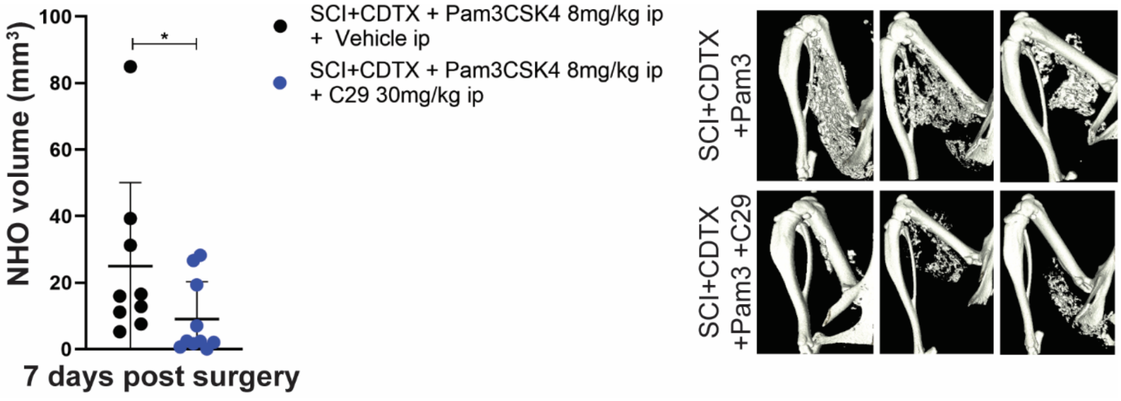

Fig. S5 TLR2 inhibitor treatment reduces SCI-NHO development in mice after Pam3CSK4 administration. C57BL/6 mice underwent SCI plus muscle injury via an intramuscular injection of CDTX (0.3125mg/kg). Mice were administered Pam3CSK4 (8mg/kg ip) after surgery and treated with either vehicle or C29 (30mg/kg) ip from day 0-3. NHO bone volumes with representative µCT images at 7 days post-surgery. Each dot represents a separate mouse, data represented as mean ± SD, * p= 0.035, two-sided Mann-Whitney.

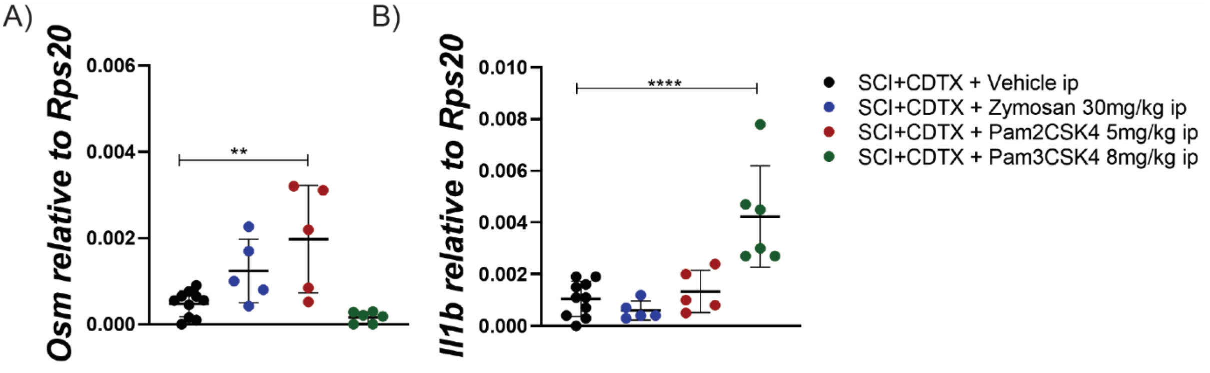

Fig S6 Administration of PAMPs increase inflammatory cytokine mRNA expression in mouse muscles developing NHO. Mice underwent SCI plus muscle injury via an intramuscular injection of CDTX (0.3125mg/kg) and were administered either Zymosan, Pam2CSK4 or Pam3CSK4 via an i.p. injection immediately after surgery. Relative mRNA expression for inflammatory cytokines (**A**) *Osm* and (**B**) *Il1b* in hamstrings of mice 20 hours post-surgery. mRNA expression was quantified relative to house-keeping gene *Rps20.* Each dot represents a separate mouse, single experiment, data represented as mean ± SD. ** $p < 0.01$, **** $p < 0.0001$.
